# Fentanyl–Antibody Interaction as a Novel Strategy against Opiates and Opioids Abuse

**DOI:** 10.1021/acs.jmedchem.4c02860

**Published:** 2025-04-03

**Authors:** Giovanni Ribaudo, Andrea Achille Taccani, Alessandra Gianoncelli

**Affiliations:** Department of Molecular and Translational Medicine, University of Brescia, Viale Europa 11, 25123 Brescia, Italy

## Abstract

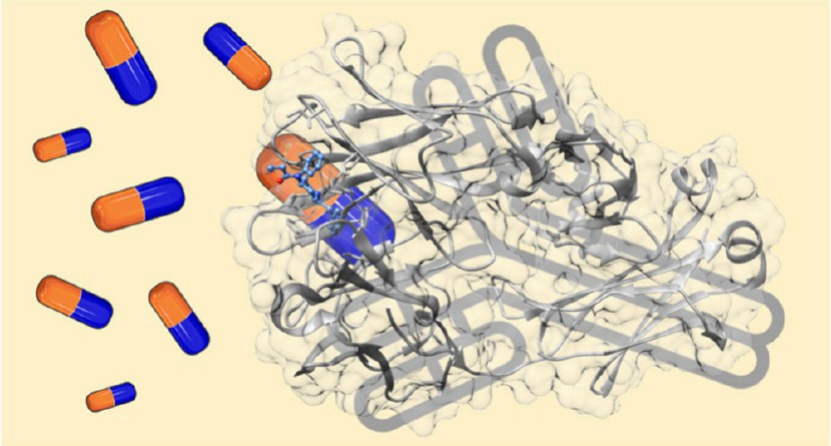

While naloxone remains *the* antidote for opioid overdoses, more efficient tools are required to effectively combat this growing crisis. Vaccines and antibodies targeting substances of abuse appear to be a novel and promising approach to tackling the fentanyl and opioid epidemic. After an initial in-depth rundown on the pharmacodynamics of the substances involved from a structural and mechanistic standpoint, and a brief overview of pharmacological approaches used in clinical settings for managing overdoses and opioid addiction, this Perspective will be mainly focused on these innovative strategies, based on the development of antibodies binding and sequestering substances of abuse and on their generation *in vivo* through vaccines. The most promising approaches will be examined, from production techniques to their potential clinical applications, analyzing the structures and mechanisms of antibody–substance interactions and comparing these with receptor binding processes.

## Significance

The opioid overdose represents an emerging social and health burden. This Perspective provides an updated overview, from the point of view of the medicinal chemist, on the pharmacological potential of an innovative approach consisting in the use of antibodies binding and sequestering substances of abuse. The two main strategies to pursue this goal, consisting in the administration of antibodies or their generation *in vivo* through vaccines, are discussed in depth.

## 1 Introduction

In today’s world, the misuse of narcotic substances, whether illegal compounds or therapeutic drugs with potential of abuse, generates increasing concerns. This problem represents a significant focus for health authorities as it annually impacts millions of individuals and families who are used to handle challenging circumstances related to substance abuse. As defined by the World Health Organization, “substance abuse” encompasses the detrimental and risky consumption of psychoactive substances, such as alcohol and illicit drugs, resulting in severe consequences for society: *“Substance abuse refers to the harmful or hazardous use of psychoactive substances, including alcohol and illicit drugs. One of the key impacts of illicit drug use on society is the negative health consequences experienced by its members. Drug use also puts a heavy financial burden on individuals, families and society”.*(1)

The misuse of psychoactive substances leads to the development of addictions and the risk of toxicity from overdose, potentially resulting in death. Substance dependence not only causes economic and social issues but also personal discomfort. Overdose fatalities prompt ethical considerations, pushing the pharmaceutical and scientific communities to seek effective interventions to prevent such occurrences. The two facets (dependence and overdose) of substance abuse are closely linked: overdoses typically happen in individuals already dependent on the substance, who, over time, build up a tolerance, leading them to escalate their doses and increasing the risk of overdose. Instances of this nature are increasing globally. According to the most recent United Nations “World Drug Report” from 2021, an estimated 296 million individuals had experimented with drugs at least once during the year. This marks an increase of about 20 million from the 2016 report’s estimate of 275 million and a substantial rise of 56 million from the 2011 report’s estimate of 240 million.^2^

While all psychoactive substances can lead to dependence, opioids pose a higher risk of fatal overdose due to respiratory depression. This burden has prompted scientific research to develop interventions for overdose prevention and treatment. Despite efforts to design opioids with reduced respiratory depression risk, challenges persist, with some synthetic opioids like fentanyl causing rapid dependence escalation and heightened overdose risk. Medicinal chemistry continues to explore ways to retain analgesic properties while minimizing harmful effects, and in particular respiratory depression.^3^ The goal is to identify alternatives to current opioids, addressing the urgent need to combat the ongoing opioid crisis.

The standard treatment for opioid overdoses, previously mainly heroin-related but now more commonly caused by fentanyl,^4^ involves μ opioid receptor (OR) antagonists. These compounds, such as naloxone and naltrexone, are listed by the WHO as essential drugs.^5^ Concerning their mechanism of action, they act as competitive antagonists by preventing the overstimulation of the μOR without activating it. Despite their effectiveness, these antagonists have limitations. The main is surely that they only address ongoing overdoses and do not prevent future ones. Administering these drugs promptly is crucial to avoid reaching the opioid concentration threshold for respiratory depression. Unfortunately, opioid overdose deaths have surged in recent years despite the availability of these life-saving treatments.^6^

The increasing use of narcotic substances and the ineffectiveness of current treatments necessitate the urgent development of a prevention strategy to address overdoses proactively rather than reactively. This challenge requires the involvement of all sectors of society, and in particular of the scientific community. In addition to public education and awareness initiatives, identifying preventive measures that leverage chemical-pharmaceutical expertise is crucial to mitigating the current alarming situation.

In the recent years, various biopharmaceutical research centers have proposed the design of preparations known as “vaccines” to stimulate the production of antibodies against narcotic substances in individuals. These vaccines aim to hinder the penetration of these substances through the blood-brain barrier (BBB), ultimately preventing psychotropic effects, acting on pleasure circuits, reducing addiction, and minimizing the risk of overdose. Indeed, this concept is not new in combating substance abuse; it was first introduced in the 1970s and it was later “re-discovered” in the early 2000s. For instance, in 2004, researchers created a vaccine known as “TA-CD” to generate antibodies against cocaine.^7^ This development, documented in the Biological Psychiatry journal, initially showed promise. However, subsequent research revealed its limited effectiveness.^8^ Consequently, it halted during phase III clinical trials and was never utilized in clinical practice.

Thanks to the increased focus on research in the field of vaccinations during the SARS-CoV-2 pandemic, along with the subsequent acquisition of knowledge and advancements in technology in this area, there is potential to leverage the established expertise in vaccine development to address the issue of drug abuse effectively. In recent years, driven by the imperative to find preventive measures, significant resources have been allocated toward the practical development of vaccine formulations for this purpose.

The aim of this Perspective is to present, from a chemical/pharmaceutical and pharmacological point of view, the current status of the utilization, particularly in combating fentanyl misuse. This article is divided into 4 main chapters which cover: introduction to opioid overdose epidemiology, structural features of fentanyl and their role in the interaction with the receptor, current strategies for treating overdose, and the emerging role of antibodies and vaccines. Particular attention was dedicated to available structural and mechanistic information. Moreover, we put our best efforts in providing the most updated results of preclinical and clinical studies. More than 110 among scientific papers, Web sites and articles were considered for the preparation of this Perspective, and graphical artworks were prepared using UCSF ChimeraX version 1.7.1. Chemical structures were produced with ACD/Laboratories ChemSketch version 2023.2.1.

### 1.1 Drug Abuse and Drug Overdoses Epidemiology

Analyzing data on the consumption, whether use or abuse, of narcotic substances globally reveals a consistent increase in the number of drug users over recent decades.

Between 1990 and 2021, based on data from the World Drug Reports by the UNODC (United Nations Office on Drugs and Crime), the global count of individuals using drugs at least once annually surged by 64.4%, rising from 180 million to 296 million. The count of individuals dependent on one or more substances also rose, from 25 million in 2003 to 39.5 million in 2021, marking a 58% increase.^9^ In the span from 2019 to 2020, the number of drug addicts escalated by 2.3 million. By comparison, during the same period, the count of cancer diagnoses surged by 19.3 million according to estimates by the World Health Organization (WHO) and the International Agency for Research on Cancer (IARC),^10^ a notably higher figure. UNODC statistics on the global distribution of substance abuse cases indicate a significant prevalence of this issue in developed nations. It is primarily a concern in first-world countries, with high percentages of drug users per capita in nations like the United States (where 19% of the population admitted to using illicit substances in 2013), Australia (14.7%), and Spain (14.4%). However, this problem has been increasingly spreading to less affluent regions in recent years.

In addition to illicit substances, the use of prescription opioids, which are opioid analgesics prescribed by physicians for pain management, is noteworthy. These medications are recognized as key contributors to the current opioid epidemic. In 2013, 5% of the American population reported using these substances.^9^ Regarding the categorization of drug users (consumers or addicts) by the substance used, data from the UNODC indicates that cannabis consumers rank highest globally in terms of user numbers, with an estimated 209 million users in 2020. Then, there are individuals who make use of opioids (61 million users), amphetamines (34 million), cocaine (21), and ecstasy (20).^9^

A distinct examination is needed for the epidemiology of deaths resulting from drug overdoses. Globally, drug overdose deaths reached approximately 600,000 in 2019 according to World Health Organization estimates.^11^ Of these fatalities, over 80% were categorized as opioid-related, where opioids were considered a contributing factor to the individual’s death but not the sole cause. Around 25% were identified as opioid-induced, meaning opioids were determined to be the primary cause of death. In comparison, 30% of drug-related deaths (equivalent to 180,000 out of the total 600,000) were solely attributed to drug use, involving substances such as opioids, amphetamines, and cocaine. Data from the Global Health Estimates in 2020 by the WHO show a significant increase in global deaths from drug overdoses over the past decade. Between 2000 and 2010, the number of deaths remained relatively stable around 100,000 worldwide. However, starting from 2010, there was a notable escalation, primarily driven by overdoses related to opioids and opiates, culminating in 180,000 deaths in 2019.^6^ In 2019, according to the same data source, out of the 180,000 reported deaths due to overdoses, approximately 100,000 were individuals aged 15 to 49, about 55,000 were between 50 and 69 years old, and nearly 25,000 were over 70. This highlights a concerning trend primarily impacting the younger and adult demographic, particularly pronounced in developed nations. Notably, the most significant rise in fatalities in recent years occurred among those aged 50 to 69, escalating from 20,000 deaths in 2000 to 55,000 in 2019, marking a 175% increase. This surge is largely attributed to the imprudent prescribing of opioids for pain relief, a practice gaining traction within this age group. Conversely, the younger and adult population often resort to illicit substances, while the elderly have long been reliant on prescribed opioids.^6^ The UNODC categorization of opioid overdose fatalities by substance indicates a predominance of heroin overdoses. However, there is a persistent and increasing occurrence worldwide of overdoses attributed to pharmaceutical opioids, specifically prescription opioids used in therapy, including fentanyl (although it is primarily illicit fentanyl that leads to fatalities, not prescribed fentanyl, as is evident).^9^ The situation is concerning, particularly in the United States of America. While drug consumption tends to be higher in developed nations, overdoses are more prevalent in North America and Oceania. Based on UNODC data from 2015, the United States exhibits a significantly elevated number of deaths per million due to drug overdoses compared to the global average: 245.8 deaths occur in the US per 1,000,000 individuals, in contrast to 39.6 worldwide. This represents an increase of over 520%.^9^ When discussing drug use epidemiology, it was noted that European countries typically rank second after North America and Oceania. However, this pattern does not hold true for overdose epidemiology: European nations stand behind Asia and Latin America in terms of deaths per population. This discrepancy can be attributed to the types of substances used (opioid consumption in Europe is not as prevalent), the availability of appropriate responses and treatments for overdoses, and the stringent European regulations that restrict opioid prescriptions, thereby impeding access and reducing the risk of addiction.^12^ Compared to the European continent, certain countries are more impacted by this issue than others. Specifically, based on data from the 2020 European Drug Report, higher fatality rates are documented in the Nordic and Anglo-Saxon nations. Sweden leads in terms of overdose fatalities with 81 deaths per million, followed by the United Kingdom (76), Finland, and Ireland (72). Upon closer examination of the statistics, Scotland stands out, surpassing American Figures in terms of number of deaths in 2018.

Apart from the specific situation in Scotland, which is indeed serious but contained,^13^ and now showing improvement since 2022, there is a pressing need for an epidemiological investigation into overdose occurrences in the United States of America. This nation stands out globally as the most impacted by this issue based on statistical data. Fatal incidents can be classified not only by sheer numbers but also by demographic factors (such as gender and age), geographical location, and notably, by the type of substance involved in the fatality, as previously addressed in the worldwide discourse on overdose epidemiology. Since 1999, when the CDC (American Center for Disease Control and Prevention) began monitoring this data, there has been a rise in the number of deaths. Initially, between 2000 and 2014, the increase was gradual and steady, followed by an almost exponential surge (mostly notable in the years 2015, 2016, and 2017).^14^ In 2018 and 2019, due to federal interventions,^15^ the death toll was contained, halting the exponential growth. However, in 2020, the overdose crisis resurged amidst the COVID-19 pandemic, resulting in record-breaking fatalities for two consecutive years: 91,799 deaths in 2020 and 106,699 in 2021.^14^ Recent projections indicate that this crisis shows no signs of abating; the measures implemented in the past two years have proven ineffective. Analyzing overdose-related deaths in the U.S. by gender, a significant predominance of male fatalities is evident, consistently doubling female deaths annually from 1999 to 2021.^16^ The 2:1 ratio of deaths between genders, which has persisted over the last two decades, highlights a substantially higher susceptibility to overdose among males. This inclination is influenced by various socio-cultural and pharmacological factors: males exhibit a greater inclination toward risk-taking behavior and easier access to drugs, coupled with limited access to medical interventions (as women are typically under closer medical supervision due to pregnancy risks). Additionally, males tend to have higher tolerance levels to substances, increasing the likelihood of consuming larger, potentially lethal doses, leading to fatal outcomes. Characterization by age of deaths reveals a gradual decrease in the age of individuals affected by overdoses, within a context of significant growth in fatalities. At the beginning of the third millennium, the majority of deaths occurred in individuals over 40 years old, encompassing both genders. However, recent data, particularly from 2020, indicates that the predominant age of those deceased is now below 40 years for both males and females. Notably, this trend is more pronounced among males, while among females, overdoses exhibit a distribution that more closely resembles a Gaussian curve.^17^ Nonetheless, statistics linking death rates to the specific substances involved hold greater significance for pharmaceutical research objectives. Overdose fatality rates remained relatively stable for approximately 15 years (from 1999 to 2014), before experiencing abrupt and concerning changes around 2014–2015. Overall, from that point onward, there has been a notable increase in deaths attributed to various substances, with the exception of heroin-related fatalities, which have shown a decline since 2016.^14^ Particularly significant rises have been observed in deaths caused by cocaine overdoses (now the third leading cause of narcotic-related deaths in the United States), amphetamine psychostimulants (primarily methamphetamine, the second leading cause of deaths in the US), and most notably, fentanyl and illicit synthetic opioids, which emerged as the primary cause of overdose deaths in 2021.^14^

### 1.2 The Shift toward Fentanyl

The escalation in fatalities related to fentanyl (and other synthetic opioids, excluding methadone) is particularly noteworthy. While the situation seemed controlled around 2011, 2012, and 2013, with 2666, 2628, and 3105 deaths respectively, the scenario shifted drastically from 2014 onward. The numbers surged exponentially: 5544 deaths in 2014 (a 78.6% increase from 2013), 9580 in 2015 (+72.8% from 2014), 18,335 in 2016 (+91.4%), and 28,466 in 2017 (+55.3%). Although there was a partial slowdown in 2018 and 2019, with 31,335 deaths (+10.1%) in 2018 and 36,359 in 2019 (+16.0%), 2020 witnessed a significant spike again (56,516 deaths: + 55.4%). The trend continued in 2021 with 70,601 deaths (+24.9%). Current estimates for 2022 and 2023 suggest a relatively stable but unresolved situation, with provisional CDC data indicating 76,226 deaths in 2022 and 74,702 in 2023 (figures updated at the time of writing), underscoring the persistent gravity of the issue.

Upon delving into the statistics, it becomes evident that fentanyl and synthetic opioids play a significant role in the rise of fatalities attributed to overdoses involving other substances, such as stimulants (cocaine and amphetamines), heroin, benzodiazepines, and antidepressants.^18^ A noticeable trend across all categories is the escalating number of deaths over the years, with the exception of heroin. Recent times have witnessed a notable shift from heroin to fentanyl addiction among drug users, owing to the widespread availability and accessibility of the latter substance. The data illustrates a clear surge in fatalities associated with cocaine and amphetamines. The majority of heroin-related deaths involve the concurrent presence of fentanyl and synthetic opioids, with minimal instances where heroin alone is the cause of death. The benzodiazepine-opioid combination can often result in a fatal outcome, as supported by the data, given the depressant effects both compound classes have on the central nervous system. While deaths related to antidepressant use are uncommon, there is a slight rise (less pronounced than with other drug classes mentioned earlier) attributed to the simultaneous use of synthetic opioids. The geographic spread of fentanyl and synthetic opioids, initially concentrated in rural areas of the Rust Belt and Appalachia, has now extended to all 50 states in the United States and is unfortunately becoming increasingly prevalent nationwide.^19^ This expansion is attributed to significant trafficking, primarily originating from China (often passing through Mexico) and to a lesser extent from Canada.^20^ Fentanyl is transported from China to Mexico either in powder form or more commonly as fentanyl precursors: 4-anilino-*N*-phenylethyl-4-piperidine (4-ANPP) and *N*-phenylethyl-4-piperidone (NPP). Fentanyl (*N*-phenyl-N-[1-(2-phenylethyl)piperidin-4-yl]propanamide) is synthesized in Mexican laboratories using these specific reagents and then shipped to the United States. These distribution channels play a crucial role in the notable rise in fentanyl availability, leading to increased consumption and subsequent fatalities. It is estimated that nearly 200 individuals in America succumb to fentanyl and synthetic opioid overdoses daily: one every 7/8 min.^21^ Fentanyl and synthetic opioids, prevalent in the American illicit market, have escalated from contributing to about 5% of overdose fatalities to 66% in 2021 and 70% in 2022. When considering deaths involving other substances where fentanyl and synthetic opioids were detected, the percentage surpasses 90%. The surge in overdoses from these substances has disproportionately impacted the youth aged 15 to 24, with fentanyl and synthetic opioids accounting for 80% of drug-related deaths in 2021, a stark increase from 6% in 2019. The increasing availability of fentanyl, including online through the dark web, has spread across rural and urban areas in North America. This concerning trend has prompted some American high schools, among others, to stock Narcan in their infirmaries.^22^ Narcan contains naloxone in an inhalation spray form and can reverse overdose effects by competitively antagonizing the μ-opioid receptor when administered correctly and promptly. Fentanyl is significantly the primary cause of death among individuals aged 18 to 45 in the United States. In 2021, fatalities resulting from fentanyl overdoses in this age group were twice as many as those from road accidents, suicides, COVID-19, heart diseases, homicides, and tumors.^14^

## 2 Chemistry and Pharmacodynamics of Fentanyl and Its Analogs

To comprehend the implications of the abuse potential of fentanyl and its derivatives, as well as strategies for prevention and treatment, an initial analysis of the chemical features of this compound family (referred to as phenyl piperidines based on their molecular structures) and the pharmacodynamic effects resulting from the interaction with opioid receptors is essential.

### 2.1 The Discovery of Fentanyl and Its Features

The history of fentanyl originates in the mid-1950s, when Dr. Paul Janssen delved into the synthesis of opioid analgesics. His goal was to produce an opioid with higher potency, not solely for a more robust analgesic effect (as existing opioids like morphine were already highly effective), but due to the belief that heightened potency would lead to increased selectivity toward the μ-opioid receptor, thus enhancing safety through lower medication doses.^23^ During that period, there was already a significant availability of opioids, enabling the production of various derivatives within this drug class. This was primarily achieved through a process of structural simplification.^24^ This process involved eliminating components not directly associated with the pharmacological effects from the original opiate structure to mitigate or eliminate the numerous side effects associated with these drugs. By starting with the morphine structure and removing the bridging oxygen that creates an epoxy ring, a group of compounds known as “morphinans” was developed. These compounds, including dextromethorphan, levorphanol, and butorphanol, retained the phenanthrene nucleus with the condensed piperidine ring (referred to as rings A, B, C, and D in the original description of opioid molecules) but omitted the E ring. Subsequently, the elimination of the C ring gave rise to another group of compounds, with the founding member being benzomorphan (thus termed “benzomorphans”), first synthesized in 1959. By removing the B ring and retaining only the A and D rings, the phenyl-piperidine family’s discovery would have been accomplished, capturing Dr. Janssen’s attention for its potent and effective qualities.

**Figure 1:**
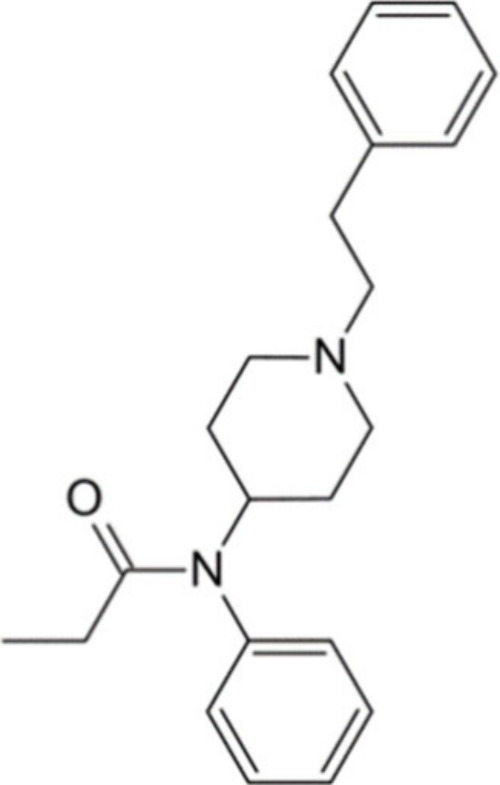
Chemical structure of fentanyl.^25^

Dr. Janssen’s team accomplished the synthesis of the fentanyl molecule in 1960, following the creation of other opioid analgesics of lower potency. Recognizing the importance of enhancing the lipophilicity of the molecule to improve its effectiveness as an opioid, Janssen modified meperidine, a synthetic opioid, by adding a phenyl substituent to the carbon linked to the piperidine nitrogen. This alteration, which increased the distance between the phenyl group and the nitrogen atom to three carbon atoms, resulted in a 20-fold increase in potency compared to morphine and a 200-fold increase compared to meperidine.^23^ Dr. Janssen refined the molecule until reaching the structure of fentanyl (Figure 1), where the ester side group was reversed from meperidine to form an amide group (with the amide nitrogen bonded directly to the carbon in position 4 of piperidine) and the distance between the piperidine nitrogen and the benzene ring was reduced to two carbons instead of three.^23^

Janssen assessed the potency of this molecule, defined pharmacologically as the amount of drug needed to produce the desired effects, to be 100–300 times greater than that of morphine. Additionally, the molecule exhibited remarkable potency and achieved a substantially higher therapeutic index (LD50/ED50: the ratio between the lethal dose in 50% of cases and the effective dose in 50% of cases, for fentanyl this ratio is 400) compared to morphine (which is 70) and meperidine (4.8).^23^

Fentanyl is therefore a synthetic opioid and the key member of the phenyl piperidine family. Its chemical structure showcases a piperidine nucleus with a phenethyl substituent attached to the piperidine nitrogen, and a propanamide linked to the carbon at position 4 of the piperidine ring via the amide nitrogen. The amide nitrogen is further substituted with a phenyl group, forming 3 bonds. With a molecular weight of 336.5 g/mol,^26^ fentanyl exhibits lipophilic properties due to the abundance of alkyl carbons and the presence of two unsaturated rings. Despite the nitrogens and amide oxygen, it has an experimental logP value of 4.05.^25^ Consequently, its water solubility is relatively low, measuring 0.74 mg/mL at neutral pH and 35 °C,^26^ and 0.2 mg/mL at neutral pH and 25 °C.^27^ Fentanyl’s p*K*_a_ is determined to be 8.43,^28^ indicating its basic nature attributed to the presence of the piperidine nitrogen.

### 2.2 The Interaction of Fentanyl with the μ-Opioid Receptor

Fentanyl interacts with ORs, which are predominantly found in the central nervous system. These receptors are not only activated by external opioids (medications or drugs like morphine, tramadol, buprenorphine, etc.) but also by endogenous opioids, peptide molecules produced by the human body that play a role in certain neuroregulatory functions. The structural aspects of the interaction of opioids with this receptor were described by Huang and colleagues.^29^ Opioid receptors belong to the G-protein coupled receptors (GPCRs) family and are classified as μOR (Mu Opioid Receptor),^30−32^ kOR (Kappa Opioid Receptor),^33^ δOR (Delta Opioid Receptor),^34^ NOR (Nociception Opioid Receptor),^35^ and ZOR (Zeta Opioid Receptor).^36^ The μ-opioid receptor is the primary opioid receptor of utmost importance from a pharmacological perspective. Research has shown that the absence or mutation of this receptor result in a complete inactivity of the entire opioid system. Despite the various components involved in this system, the μ-opioid receptor is deemed essential for its activation.^37,38^ Fentanyl primarily targets the μ-opioid receptor, but involvement of κ-OR and δ-OR is also described.^39^ Currently, novel analogues with selectivity for a receptor subtype are being studied.^40^ Additionally, it must be also considered that fentanyl is among opioids that, while presumably acting primarily at the same single receptor, activate distinct downstream responses. This phenomenon is known as “functional selectivity”^41^ and defines the pharmacological profile of the drug. In the case of fentanyl, pharmacological outcomes consist in analgesia and sedation, along with significant side effects, notably respiratory depression. Failure to address this can lead to severe consequences like apnea and patient fatality.

**Figure 2:**
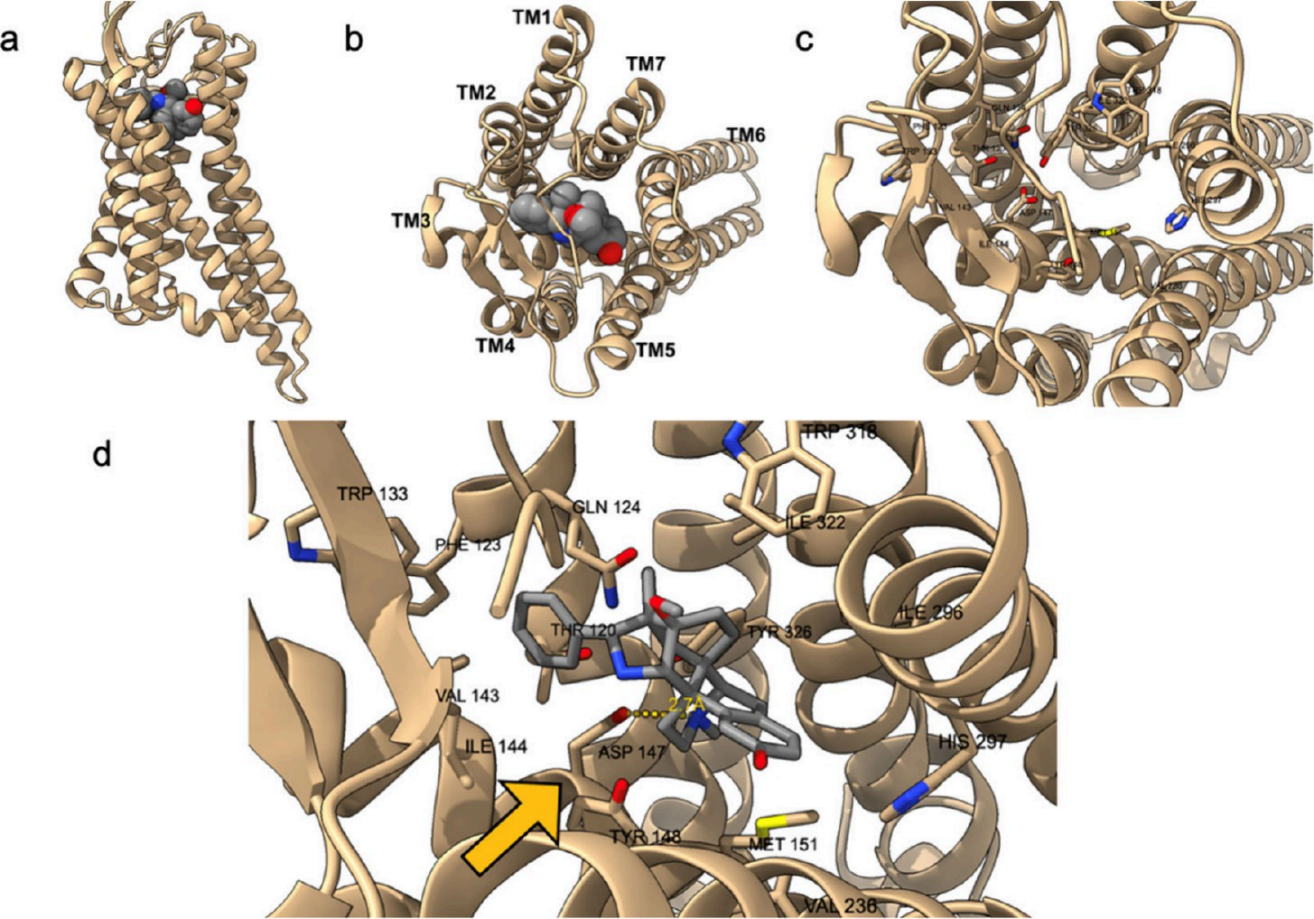
Structure of the μ-opioid receptor–BU-72 complex from various perspectives (a–c; a detailed view of the binding pocked is depicted in panel d).

The high-resolution structure of the μ-opioid receptor binding pocket has been elucidated using X-ray crystallography^42^ and cryo-electron microscopy techniques.^43^ The PDB entry 5C1M shows the interaction with the receptor of BU-72, an investigational morphinan ligand used for research purposes due to its high potency.^44^ In Figure 2, panel “a” displays a side view of the receptor-ligand complex; panel “b” shows a top view; panel “c” reveals the binding pocket of the μOR from above without the ligand present; and panel “d” provides a close-up of the BU-72-μOR bond.

Thus, the binding pocket for opioid molecules lays between the transmembrane (TM) domains TM3, TM5, and TM6. Residues of TM2 and TM7 also influence binding, facilitating ligand interaction. In contrast, TM1 and TM4 domains are not directly involved in the binding process. Opioid ligands, such as BU-72 and DAMGO, exhibit rigidity when trapped in the pocket, as evidenced by molecular dynamics simulations. The mobile part of both molecules is the portion facing TM3, specifically the “fifth residue” (Gly-ol substituent). A critical interaction occurs between the ligand’s positively charged nitrogen and the aspartic acid carboxyl group of the conserved residue Asp147 in TM3. For BU-72, the distance between the tertiary amine and aspartate carboxyl is 2.7 Å. Additionally, the ligands interact with the imidazole ring of a conserved histidine at position 297 (His297) on TM6 through hydrogen bonds mediated by water molecules. Studies suggest that mutations at positions Asp147 or His297, or lower pH levels affecting His297 protonation, decrease ligand affinity for the μOR. The presence of conserved Asp147 and His297 is crucial for establishing bonds with DAMGO or BU-72, as observed in the reported structures.

Several studies have been conducted in recent years to investigate the binding pose of fentanyl and other opioids within the μ-opioid receptor binding pocket. The aim is to comprehend the mechanism of μOR activation by this compound family and the structure–activity relationship (SAR) of fentanyl and its derivatives. Molecular docking and molecular dynamics (MD) simulations in the literature consistently highlight the crucial interaction of all opioids with Asp147 (sometimes noted as Asp149). This interaction involves the formation of a salt bridge between the negatively charged carboxyl residue of Asp147 at physiological pH and the positively charged nitrogen of the opioid molecules, such as the nitrogen of the piperidine ring in fentanyl. The results of the simulations suggest that in the well-characterized binding pocket of the μ-opioid receptor, the fentanyl molecule predominantly forms a salt bridge with Asp149 (or 147) and occasionally can bind deeper by forming a hydrogen bridge with His299 (or 297).^45^ Additionally, the simulations indicate that fentanyl is more inclined to adopt the “APF” configuration within the pocket, with the amide positioned deeply, the piperidine in the middle, and the phenylethyl on the surface, featuring the amide in cis conformation and His299 of the μ-opioid receptor in the δ tautomer.^46^

In November 2022, the structure depicting the fentanyl molecule bound to the μ-opioid receptor was successfully resolved for the first time utilizing cryo-EM.^47^ The findings were afterward documented in Protein Data Bank (PDB ID: 8EF5).^48^

The research conducted by Zhuang et al.^47^ resulted in the discovery that the fentanyl molecule adopts a “Y” conformation within the orthosteric pocket. This conformation involves the piperidine ring and the phenylethyl forming the base of the Y, while the aniline ring and the amide group constitute the arms. The fentanyl molecule spans residues TM2, TM3, TM6, and TM7 of the transmembrane domain, confirming the APF pose with the amide in the cis configuration as previously simulated. In this structure, fentanyl positions the phenylethyl moiety towards the cleft of TM2 and TM3, and the propionyl group towards TM6, engaging in weak hydrophobic interactions with the alkyl side chains of Ile298 and Val302. The nitrogen-bound aromatic ring (aniline) faces the core of the transmembrane region, establishing a network of hydrophobic interactions with the side chains of Met153, Trp295, Ile298 on TM6, Gly327 and Tyr328 on TM7. A significant bond is the salt bridge between the piperidine nitrogen of fentanyl and the carboxylate of Asp149 on TM3, a common feature among opioids binding to ORs. Additionally, Asp149 on TM3 interacts polarly with neighboring amino acids: Gln126 on TM2 and Tyr328 on TM7. This network, known as the DQY motif, is conserved among ORs and plays a crucial role in OR activation. The benzene ring linked to the amide nitrogen of fentanyl establishes direct π-π interactions with Trp295 on TM6 and Tyr328 on TM7. Notably, the phenylethyl of fentanyl occupies a concealed hydrophobic pocket between residues TM2 and TM3, enhancing binding stability. Mutations near this minor binding pocket reduce receptor activation. Trp150 and Met153 on TM3, Val238 on TM5, Ile298 and His299 on TM6 are also involved in fentanyl binding, with mutations in these areas leading to diminished G protein and β-arrestin signaling. While the structure by Zhuang et al. suggests that the conformation of fentanyl does not significantly impact histidine tautomerism, the potential influence of tautomerism on other binding poses cannot be disregarded due to cryo-EM capturing only one binding pose.

When comparing the binding mode of fentanyl with that of morphine to the μ-opioid receptor, it is evident that morphine presents a more condensed structure than fentanyl, taking on an elliptical “O” shape. It interacts with the same hydrophobic amino acids on TM3, TM6, and TM7. The morphine nucleus aligns with the propionyl group of fentanyl, while the hydroxyl of the phenolic part of morphine faces TM5. The amino group of the D ring of morphine establishes a salt bridge with the carboxylate of Asp149, similar to the amine of piperidine in fentanyl.

**Figure 3:**
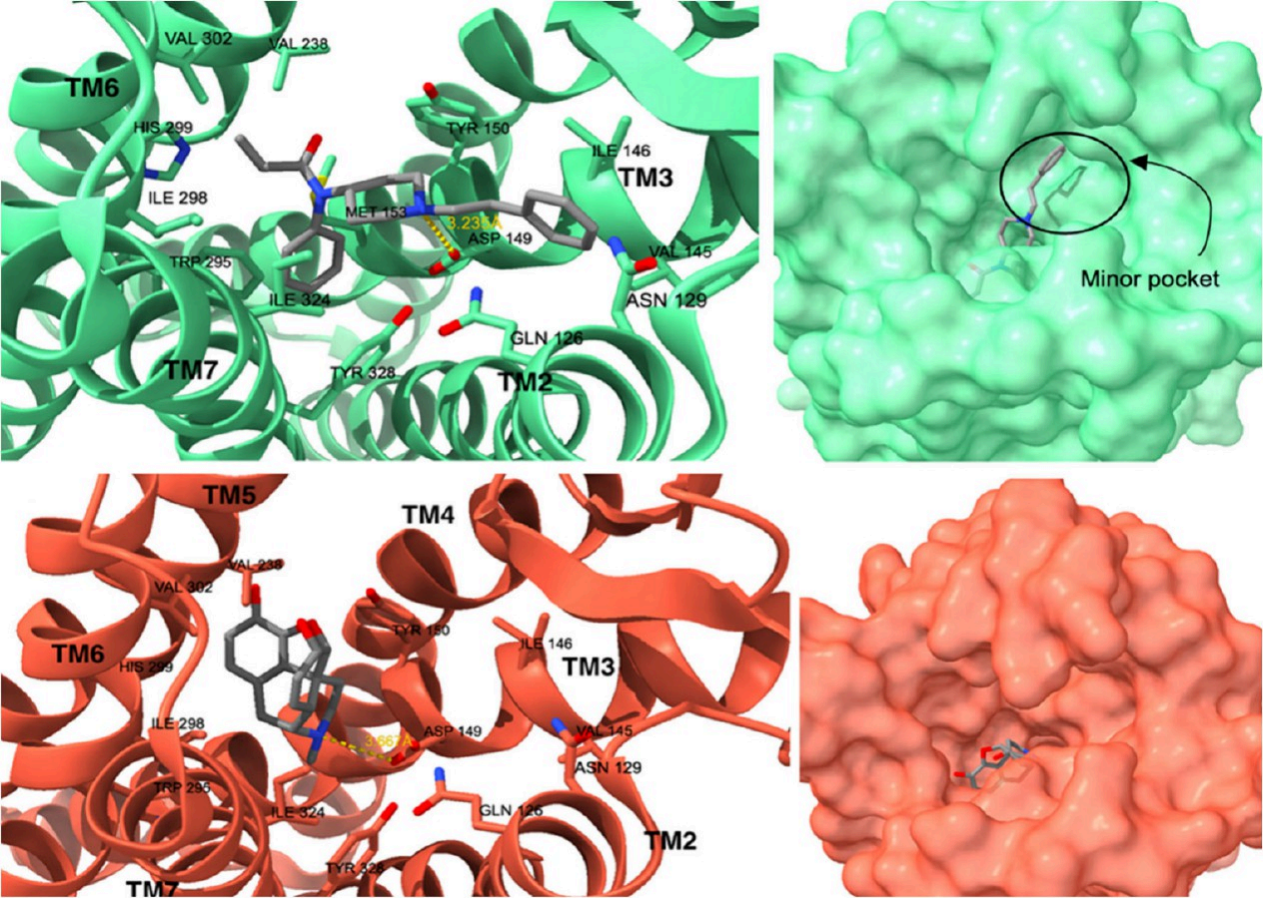
Fentanyl−μOR complex (PDB ID: 8EF5; μOR in green) alongside the morphine−μOR complex (PDB ID: 8EF6; μOR in red).

Morphine, due to its smaller and clustered structure, lacks the ability to engage in interactions comparable to those facilitated by the phenylethyl moiety (morphine cannot access the concealed “secondary” pocket) and the aniline ring of fentanyl. This difference elucidates the pharmacodynamic rationale behind the heightened potency of fentanyl, which can trigger μOR with a potency 50–100 times greater than morphine at equivalent doses, even though, actually, the exceptional lipophilicity of fentanyl, enabling it to effortlessly traverse the BBB, primarily underpins this phenomenon.^49^ The binding motif is depicted in Figure 3, contrasting the positions of fentanyl and morphine in the orthosteric pocket of the μ-opioid receptor. Fentanyl exhibits an affinity constant for μOR (K_μ_) of 1.2 nM^50^ and this strong binding is granted by a network of interactions resumed in the following: hydrophobic interactions with Ile298, Val302, Met153, Trp295, Ile298, Gly327 and Tyr328, a salt bridge with Asp149, π-π interactions with Trp295 on TM6 and Tyr328 and other interactions with Trp150 and Met153 on TM3, Val238 on TM5, Ile298 and His299. In Figure 3, panels “a” and “c” provide a detailed view of the orientations of fentanyl and morphine, respectively, highlighting the compact nature of morphine in comparison to fentanyl. Panels “b” and “d” illustrate how fentanyl can access a “minor” orthosteric pocket by leveraging the extension of phenylethyl moiety.

### 2.3 Activation of the μ-Opioid Receptor

**Figure 4:**
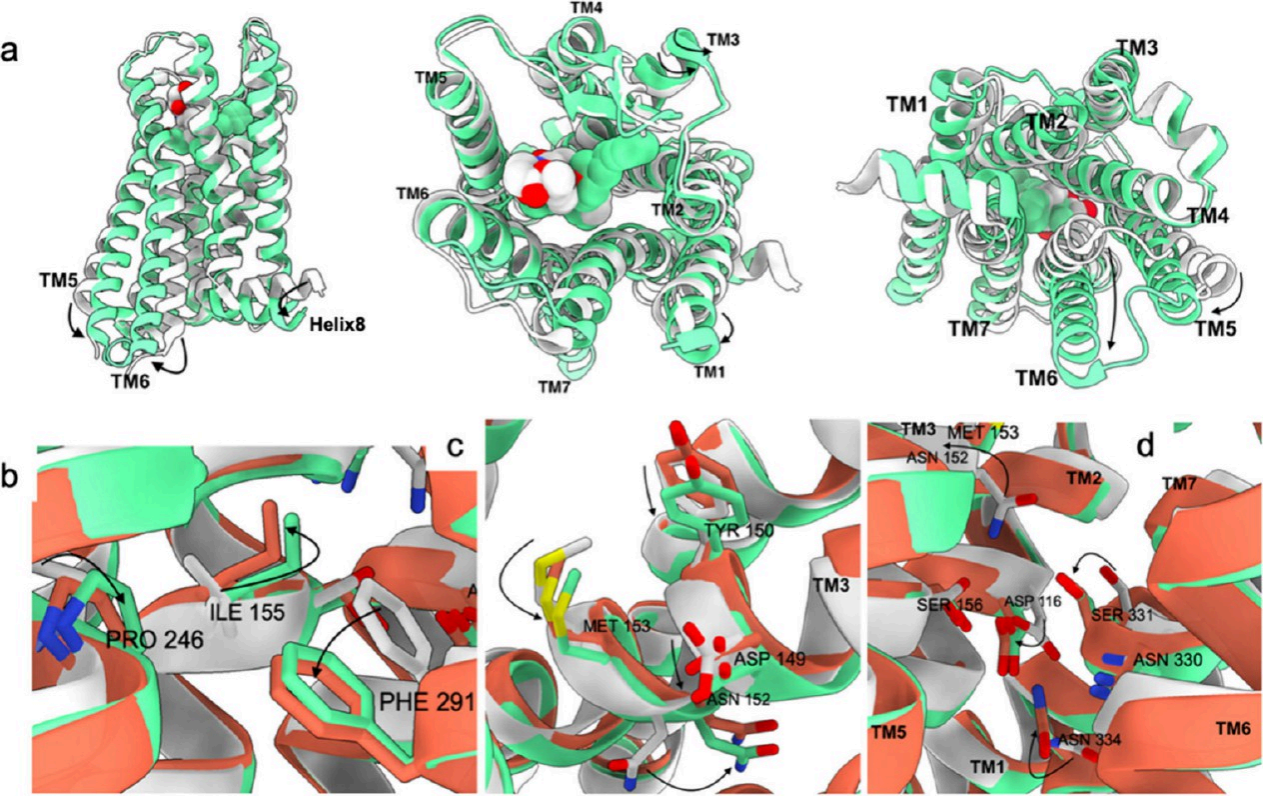
Conformational changes induced by fentanyl activating the μ-opioid receptor. The active μOR is depicted in green (fentanyl-bound) and red (morphine-bound), while the inactive form is shown in white (β-FNA-bound). The Figure includes a general view (a), a focus on the PIF motif (b), on the DYYNM motif (c), and an illustration of the impact of N152 movement (d).

The cryo-EM structures elucidated by Zhuang et al.^47^ reveal notable structural variances (see Figure 4) between the active state of μOR interacting with fentanyl) and the inactive state (interacting with β-FNA, an antagonist): in the active state, the third transmembrane domain TM3 undergoes a twisting motion; TM5 shifts inward while TM6 moves outward; TM2 and TM7 undergo a clockwise rotation. The hydrophobic interaction between the aniline group of fentanyl and Trp295 of TM6 leads to a 28° rotation of tryptophan, orienting toward Phe289 (also located on TM6), which in turn moves away and rotates; consequently, the neighboring Ile157 of TM3 and Pro246 of TM5 also alter their conformation, approaching Phe289 due to favorable interactions. Binding with Asp149 triggers a rotation of the entire DYYNM motif (Asp149, Tyr150, Tyr151, Asn152, Met153, all on TM3 in sequence), leading to a twisting of the whole TM3. Mutations at Asn152, which leads to a persistent μOR activation, confirms the significance of this conformational alteration. These movements induce a reorganization of certain motifs, causing TM6 to shift outward and TM7 and TM2 to rotate. Despite distinct binding orientations of fentanyl and morphine, the induced conformational change in the receptor remains similar. These findings put the bases for future research in this direction, and in the last year several papers appeared in the literature focused on the role of conformation and chirality, also from a dynamic point of view.^51−56^

Recent research revealed that the activation of μOR, like other GPCRs, entails a dual downstream signaling process: the signaling pathway associated with the Gi protein activation and the pathway mediated by β-arrestin. The desired pharmacological effects, such as analgesia and sedation, are induced by the G protein pathway activation, while the adverse effects are linked to β-arrestin activity. β-arrestin is accountable for side effects like respiratory depression, constipation, nausea, and vomiting. Conventional opioids, including fentanyls, typically trigger both pathways equally. As discussed in a previous section, medicinal chemists are investigating molecules capable of segregating the analgesic effects from the side effects by selectively activating the Gi protein pathway, thereby diminishing adverse reactions.

In this context, Zhuang et al.^47^ proposed that interactions with the TM6 and TM7 domains prompt the activation of the β-arrestin pathway.

**Figure 5:**
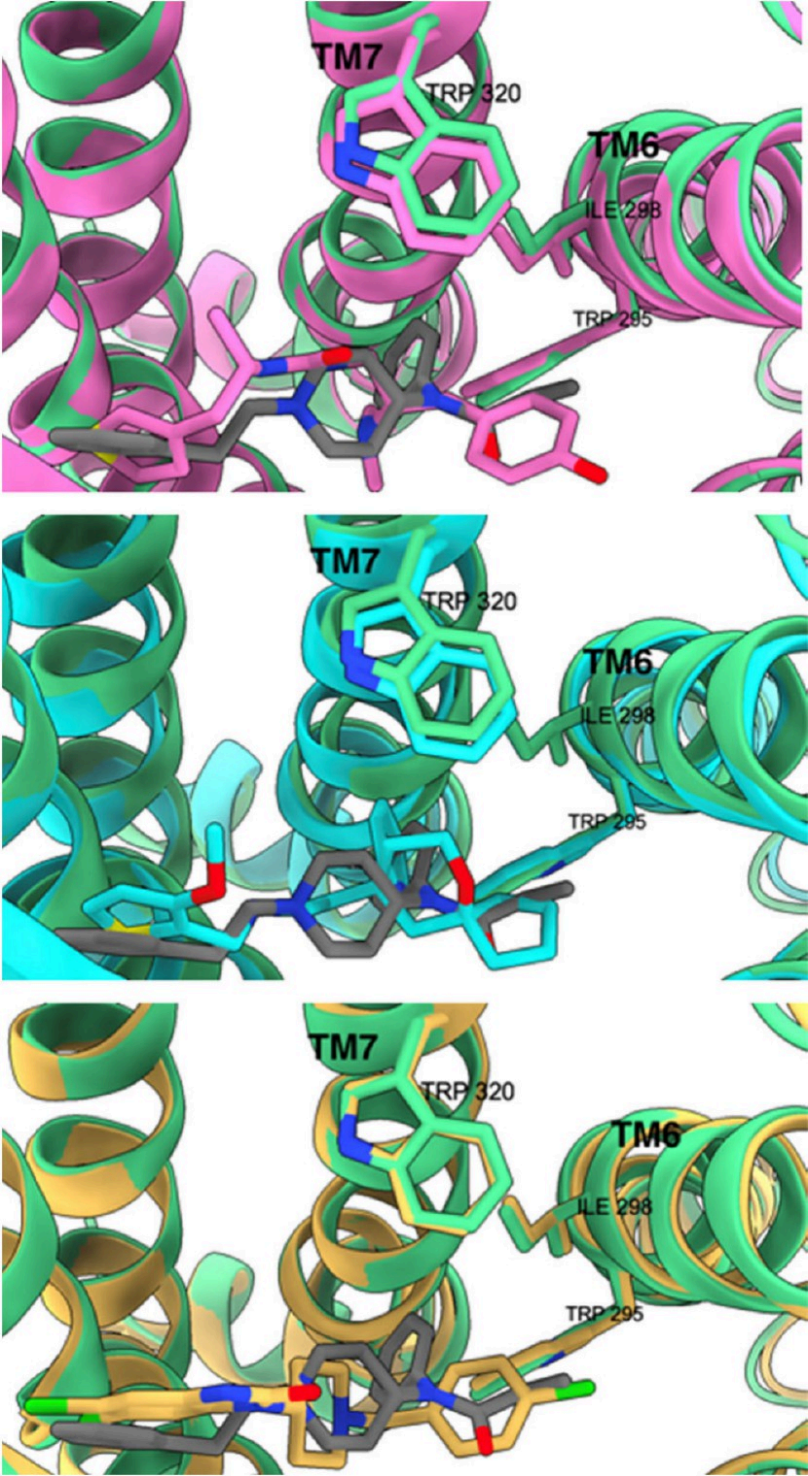
From top to bottom; binding of PZM21 (pink) and fentanyl (gray) to the activated μOR (pink, by PZM21, green, by fentanyl); TRV130 (light blue) and fentanyl (gray) bound to the activated μOR (light blue, by TRV130, green, by fentanyl); SR17018 (yellow) and fentanyl (gray) bound to the activated μOR (yellow, by SR17018, green, by fentanyl).

SR17018, TRV130, and PZM21 are μ agonists that exhibit minimal or no activation of the β-arrestin pathway in comparison to fentanyl (PDB IDs: 8EFL, 8EFB, 8EFO; Figure 5). These three compounds in the binding pose appear to be distanced from TM6 and TM7, residing in the orthosteric pocket above Trp295 of TM6. SR17018 and PZM21 do not engage with TM7 residues; SR17018 shifts 1 Å away from TM7 toward TM3 in contrast to fentanyl. SR17018 and PZM21 establish weak hydrophobic interactions solely with the TM6 domains.

The flexible and mobile chlorobenzene moiety of SR17018 facing TM6 implies instability in that region (unlike fentanyl and morphine, which exhibit rigidity in the corresponding areas). TRV130 demonstrates slightly higher β-arrestin activation than SR17018 and PZM21, albeit still at a low level. The binding pose is similar; TRV130 forms more hydrophobic interactions with TM6 and TM7 compared to SR17018 and PZM21, yet these interactions remain less efficient than that of fentanyl (the pyridine of TRV130 is positioned 35° farther from TM6 and TM7 than the aniline of fentanyl). Additional support for the theory that binding with TM6 and TM7 triggers the β-arrestin pathway is derived from simulations involving the binding of fentanyl, morphine, and DAMGO to the μOR with amino acid residues of TM6 and TM7 typically employed in mutated binding scenarios: the presence of mutated Trp295, Ile298, and Trp320 significantly diminishes β-arrestin activation while also the activation levels of the G protein slightly decrease.

### 2.4 Fentanyl Derivatives: Structure–Activity Relationship and μ-Opioid Receptor Binding

The fentanyl molecule has been a model for a whole generation of compounds, referred to as fentanyl analogs, or fentalogs. In fact, by exploring the SAR, numerous derivatives have been developed since 1964. Despite similarities to phenylpiperidines, from which fentanyl originates, and morphine-based opioid derivatives, fentanyl’s pharmacophore is anilidopiperidine rather than phenylethyl.

There are numerous possibilities for modifying the molecule: replacing the aniline with another heterocyclic ring while retaining aromaticity or using nonaromatic groups; converting the amide nitrogen into a carbon; combining the propionyl group with the aniline in the ortho position or changing it into another acyl substituent; substituting the carbonyl oxygen with sulfur to create a thiocarbonyl or with carbon to form a methylene; substituting the piperidine ring in the free positions or transforming it into other heterocycles or to linear analogues. Substituents can be added to phenylethylbenzene, or the ring can be altered to convert it into a heterocycle; finally, introducing substituents to the two ethyl carbons. Vardanyan and Hruby collected and analyzed the most important derivatives synthesized over the years, many of which were reported by Janssen Pharmaceutica, in a review of crucial importance.^57^

Among the most important fentalogs, substituents to the aniline are seldom seen, with only brifentanil and trefentanil featuring a fluorine in the ortho position of the aniline ring. Mirfentanil stands out for having a heterocycle instead of an aromatic ring composed solely of carbons, indicating limited potential for enhancements to the ring. The aromatic ring resides in the orthosteric pocket close to positively charged residues of the μ-opioid receptor. Bulky structures displace fentanyl from the binding site, with only substituents like fluorines enhancing receptor affinity. Substituents located at carbon 3 relative to the piperidine nitrogen in the fentanyl structure, typically weaken the bond due to unfavorable interactions with receptor amino acid residues. Conversely, at carbon 4 of the piperidine ring, groups can significantly boost affinity for the μOR. Examples include the methoxymethyl fragment in sufentanil and alfentanil, the 4-methylcarboxylate in carfentanil, lofentanil, and remifentanil, or the benzene in brifentanil and trefentanil. These lipophilic substituents facing the propionyl side enhance overall lipophilicity, aiding BBB penetration and strengthening binding affinity through favorable receptor interactions. Carfentanil, the most potent opioid, surpasses morphine’s analgesic potency by 10,000 times^58^ and is solely used in veterinary anesthesia. Carfentanil exhibits immense affinity for the μOR (K_μ_ = 0.024 nM; compared to fentanyl’s K_μ_ = 1.2 nM).^50^ Sufentanil, the most potent opioid for human use, is 5 to 10 times stronger than fentanyl or 500–1000 times more potent than morphine.^59^ Notably, substituents at carbon 3 or 4 of the piperidine ring can impact the molecule’s chirality, with fentanyl being achiral while its derivatives gain chirality from substituents at these positions. Substituents adjacent to the piperidine nitrogen minimally impact compound activity. Brifentanil and mirfentanil exhibit modifications in the acyl portion from the original fentanyl structure; typically, lipophilicity is necessary in this position to match the hydrophobic nature of amino acid side chains in the fentanyl propionyl orthosteric pocket. The piperidine N-substituent must be sufficiently extended and flexible to access the “minor” binding pocket of the μ-opioid receptor. These groups in fentanyl derivatives closely resemble phenylethyl properties; phenylethyl is conserved in most fentalogs. α-Mefentanil features a methyl in α to the piperidine nitrogen, while OH-mefentanil has a hydroxyl in β. Sufentanil contains an ethylthiophene; alfentanil, along with brifentanil and trefentanil (not used clinically), has a dihydrotetrazole at the end of the ethyl chain, with a carbonyl as the sole carbon and an ethyl residue replacing the nitrogen linked to the carbonyl. Alfentanil’s structure results in a pKa of 6.5, allowing it to rapidly cross the BBB due to its non-ionized state in biological fluids. These derivatives, known as short-acting fentanyls, have a rapid onset of action but carry a heightened risk of respiratory depression, limiting their clinical use. Remifentanil stands out with an ester in this position, setting it apart from other derivatives. It boasts a potency 100–200 times greater than morphine but has a brief duration of action due to tissue esterases converting it into less active forms. Its short half-life makes it suitable for anesthesia, with transient and manageable side effects. However, its rapid metabolism precludes its use as an analgesic.

As can be deduced from the overview presented above, the SAR of fentanyl and its derivatives has been widely studied through the years. While the detailed discussion of these aspects is out of the scopes of this Perspective, the interested reader can refer to other studies in the literature for more focused papers.^60−62^

Molecular docking simulations of significant derivatives (carfentanil, lofentanil, sufentanil, remifentanil, and ohmefentanil) were conducted by Zhuang et al.^47^ using the Schrodinger Glide software. The examination of the binding of these molecules to the μ-opioid receptor provides a detailed insight into their SAR. The binding orientation of all derivatives was observed to closely resemble that of fentanyl, albeit with minor structural variances that play a crucial role in determining potency differences. Carfentanil, in comparison to fentanyl, establishes hydrophobic interactions with Ile298 of TM6, as well as with Trp320 and Ile324 of TM7, facilitated by the methoxycarbonyl group at position 4 of the piperidine ring. This interaction enhances the binding to the μOR, elucidating the heightened potency. Sufentanil and remifentanil also engage in interactions akin to those of carfentanil with TM6 and TM7 residues; alterations at Ile298, Trp320, and Ile324 notably diminish the binding affinity of these derivatives. Lofentanil and ohmefentanil, along with fentanyl, establish a hydrophobic interaction with Tyr150 of TM3.

The above-mentioned structural information derived from SAR studies, structural determination of fentanyl-receptor complexes and molecular simulations, were essentials to design compounds with limited impact on β-arrestin pathway, and thus with more limited side effects. This approach will be discussed in the following section.

### 2.5 Novel Derivatives that Avoid Stimulation of the β-Arrestin Pathway

A novel strategy pursued in medicinal chemistry involves separating the activation of the desired pathway mediated by the Gi protein from the activation of the β-arrestin pathway, which is responsible for the dangerous side effects associated with the opioid epidemic. Zhuang et al.^47^ not only confirmed that the activation of the β-arrestin-mediated signaling results from ligand interactions with specific amino acid residues in the TM6 and TM7 domains, but also proposed fentanyl derivatives with reduced interactions with TM6 and TM7 to selectively activate the Gi pathway. By replacing the aromatic ring with an isopropyl or propyl group linked to the amide nitrogen, the aniline group in fentanyl, responsible for interactions with TM6 and TM7, was modified in the proposed molecules. Additionally, a methyl group was introduced at the 3-position of piperidine, and a fluorine atom was placed para to the phenyl group connected to the piperidine nitrogen via an ethyl group. Two compounds, named FBD1 and FBD3, were obtained through the synthetic approach. Molecular docking simulations conducted using the Maestro software from Schrodinger revealed that FBD1 and FBD3 exhibit similar binding poses, with the propyl substituent of FBD3 penetrating deeper than that of FBD1. This disparity may explain why FBD3 induces β-arrestin recruitment at higher rates compared to FBD1, as the propyl group of FBD3 extends further toward TM6 and TM7, engaging in hydrophobic interactions with Trp295 and Tyr328.

The involvement of this mechanism was also investigated by Tsai and colleagues in very a recent paper, in which the authors studied the efficacy of fentanyl and nitazene analogs in the β-arrestin signaling.^63^ On the other hand, Yamaguchi et al. studied the selectivity of nalurafine analogs (κ-OR agonists) for G protein- and β-arrestin-mediated pathways.^64^

The reduced stimulation of the β-arrestin pathway could represent a significant advancement in the clinical management of pain, and these molecules, or similar ones, may represent a substantial leap forward compared to the compounds presently utilized in clinical settings. Over time, utilizing molecules that differentiate the activation of the Gi protein pathway from the activation of the β-arrestin pathway, if proven effective in vivo without unexpected side effects, could potentially replace the use of traditional opioids. Nevertheless, in the short term, these compounds are not a solution to the epidemic caused by fentanyl. The high number of overdoses are due to the misuse of the substance as an illicit drug rather than to its controlled clinical application.

After comprehending the chemical properties of fentanyl and its derivatives, examining their binding to the primary target, the μ-opioid receptor, understanding their activation mechanism, and identifying the biochemical causes of adverse effects, the next section will cover the pharmaceutical research solutions and current clinical practices aimed at mitigating these effects, with a primary focus on addressing addiction and overdose.

## 3 Current Treatment of Overdose and Dependence by Fentanyl and Derivatives

### 3.1 Overdose and Opioid Use Disorder

The unregulated utilization of fentanyl and its derivatives entails both the immediate danger of experiencing an overdose and the long-term risk of developing an addiction, which is marked by the common symptoms of opioid use disorder (OUD). The significant abuse potential of fentanyl and its derivatives primarily stems from their pharmacokinetic properties. These lipophilic molecules can induce pharmacological effects within minutes or even seconds of administration. While this rapid onset is advantageous in fields like anesthesia, where quick action is crucial, it also makes these compounds appealing for recreational use due to the rapid onset of euphoria, gratification, and pain relief. The high logP value of fentanyl, surpassing that of heroin,^65^ allows anilidopiperidines to rapidly cross the BBB and bind to μ-opioid receptors in the central nervous system, setting them apart in the realm of opioids. Alongside their rapid therapeutic effects, these compounds can quickly trigger side effects, with respiratory depression being the most concerning. This condition, characterized by decreased respiratory rate and depth, often accompanied by a condition called “wooden chest syndrome”,^66^ can lead to severe complications like hypoxia and, in worst-case scenarios, death by asphyxiation. The likelihood of experiencing these adverse effects is dose-dependent, with higher doses increasing the risk of overdose toxicity. While the abuse potential primarily lies in pharmacokinetics, the probability of side effects is also influenced by pharmacodynamics. Fentanyl and its derivatives exhibit a higher affinity for μ-opioid receptors compared to other opioids, resulting in a lower potentially lethal dose. The Drug Enforcement Administration (DEA) indicates that a lethal plasma dose of fentanyl for an average 70 kg adult is 2 mg (0.03 mg/kg),^67^ with doses exceeding 0.025 mg^68^ posing a significant risk of death in unmonitored breathing environments. Although the lethal doses of derivatives remain unknown, they are likely within similar ranges. Carfentanil, a potent derivative, is estimated by the DEA to have a lethal dose 100 times lower than fentanyl due to its increased potency, equal to 2 μg for a 70 kg adult (0,03 μg/kg). For comparison, the lethal plasma dose of heroin for a 70 kg adult is 100 mg.^69^ The DEA confiscated a record 74.5 million fentanyl tablets in 2023, significantly exceeding the previous record of 58.3 million in 2022. Laboratory analyses revealed that, on average, 7 out of 10 seized tablets contained a lethal fentanyl dose. Consequently, the DEA launched the “One Pill Can Kill” media campaign^70^ to increase awareness of the dangers of fentanyl consumption. Similar to substances like MDMA, fentanyl can be deadly even in small quantities, as there is a high likelihood of encountering tablets with fentanyl doses exceeding 2 mg. A study published in the British Journal of Pharmacology^71^ examined the extent of fentanyl overdose compared to heroin and morphine. To eliminate variability from oral administration, the study focused on values from parenteral administration. Findings revealed that fentanyl is about 70 times more potent in inducing respiratory depression and acts much faster. Just 2 min after fentanyl injection, individuals experience breathlessness with decreased tidal volume and respiratory rate. Respiratory depression from fentanyl occurs much quicker than with morphine and heroin for equipotent doses. For instance, administering 1 mg of fentanyl results in faster respiratory depression than 100 mg of morphine or 50 mg of heroin. Fentanyl significantly reduces tidal volume, unlike morphine and heroin, which mainly lower respiratory rate. Consequently, all three substances decrease minute ventilation, but fentanyl causes a more pronounced and rapid reduction. In addition to the risk of overdose, whose biochemical reason lays in the activation of the β-arrestin pathway by fentanyl, that reduces respiratory rate, the drug also induces euphoria and gratification, leading to psychological and physical dependence. Over time, tolerance to the effects of the drug develops, necessitating higher doses for the desired outcome. These factors contribute to OUD, increasing the risk of fatal overdose.

### 3.2 Naloxone: Chemical and Pharmacodynamic Properties

**Figure 6:**
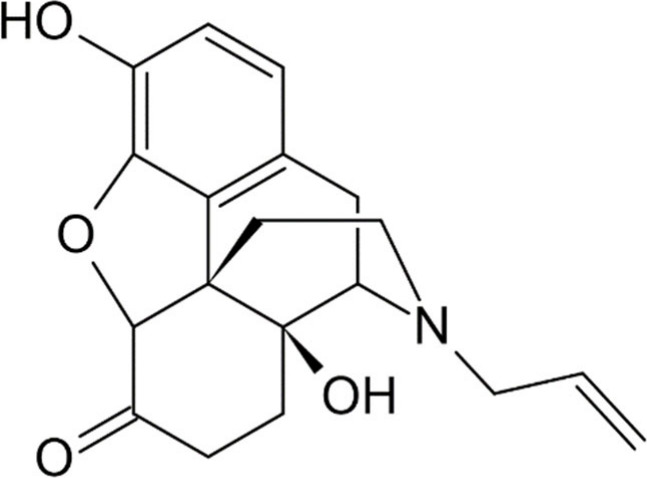
Chemical structure of naloxone.^72^

The unregulated Naloxone (IUPAC name (−)-17-(allyl)-4,5-epoxy-3,14-dihydroxymorphinan-6-one, Figure 6)^72^ is a semisynthetic opioid derived from the alkylation of 14-hydroxydihydronormorphinan with allyl bromide at the amino nitrogen located within the D-ring of the morphine structure.^73^

Before the introduction of naloxone, nalorphine, and levallorphan were utilized for managing respiratory depression. However, these molecules, when administered independently, could induce respiratory depression by themselves.^74^ Naloxone, in its hydrochloride form, was developed in the 1960s as the first μ-opioid receptor antagonist capable of mitigating opioid-induced respiratory depression without eliciting agonist effects on the OR. FDA approval was granted in 1971, establishing it as the most commonly used agent for reversing opioid overdoses. Initially, naloxone was predominantly administered in hospital settings by trained clinical professionals. In 1996, its distribution expanded to the community through the Chicago Recovery Alliance.^75^ Nalorphine, levallorphan, and naloxone share structural similarities as antagonists, all being derivatives of morphine. Naloxone, in comparison to morphine, maintains the structure of five condensed rings, the hydroxyl group in position 3, and the epoxy bridge. The hydroxyl at position 6 in morphine is oxidized to a carbonyl in naloxone. Additionally, naloxone features an extra hydroxyl at position 14 and lacks the double bond between C7 and C8 present in morphine. The primary alteration lies in the substitution of an allyl for a methyl to replace the amino nitrogen, a distinguishing characteristic shared by nalorphine and levallorphan, defining their role as antagonists.

The essential role of N-allyl in the binding pose has been elucidated through molecular dynamics simulations comparing the binding dynamics of naloxone with morphine within the μ-opioid receptor. Despite the similar structure, the initial binding pose within the orthosteric pocket of the μOR is analogous. However, as demonstrated by Zhang et al. in a simulation (conducted using the IFD approach, or flexible induced fit through Schrodinger Desmond software) published in Frontiers, the allyl of naloxone functions as an “anchor” that stabilizes its movement by limiting its rotational freedom, thereby reinforcing the binding pose.^76^

Although morphine and naloxone share the same molecular scaffold, they exhibit distinct behavior in the orthosteric pocket of the μ-opioid receptor. Both molecules bind to Asp147, interact with Ile322 and Tyr326, albeit differently. Naloxone additionally interacts with the residues Glu229, Lys233, and Val236, which morphine does not engage with. The primary difference lies in the rigidity of the structure: the allyl moiety of naloxone attaches to Ile322, restricting movement solely around it. In contrast, morphine is more mobile, allowing for deeper positioning. A validation of the significance of the interaction between the allyl group of naloxone and Ile322 is evident in a binding simulation with a μ-opioid receptor containing the mutated isoleucine. When the size of the Ile322 side chain is reduced by converting it to an alanine, naloxone can penetrate further into the binding pocket and function as an agonist. Unlike the methyl at position 17 of morphine, which cannot stably reach Ile322, the allyl group of naloxone impedes its movement, affecting not only the small molecule itself but also the TM7 and TM6 domains. From the point of view of the molecular mechanism, this hindrance prevents the conformational changes necessary to activate the G protein and β-arrestin pathway.

Naloxone acts as a competitive inhibitor for the μ-opioid receptor, displacing the ligand to prevent receptor activation. While effective in opioid overdose cases, its efficacy against fentanyl and derivatives has been questioned in reports published by the CDC^77,78^ The potency and pharmacokinetics of synthetic opioids often necessitate higher naloxone doses for restoring normal breathing. Naloxone is administered intravenously in hospitals in the US, while intranasal spray is the most common preparation out of these structures. Narcan IN spray, approved by the FDA in 2015, has a dosage of 4 mg/0.1 mL, and often requires repeated administrations. To address this, higher-dose products like KLOXXADO (8 mg/0.1 mL, IN spray) and ZIMHI (5 mg, IM or SC) were approved by the FDA in 2021. Concerns have been raised about the use of higher doses due to the risk of paradoxical opioid withdrawal symptoms and pulmonary edema.^79,80^ Additionally, unintended reductions in patient acceptance were also reported. In their very recent article, Lemen and colleagues “do not recommend high-dose naloxone formulations as a substitute for four doses of IM or IN naloxone due to the higher cost, risk of precipitated withdrawal, and limited evidence compared to standard doses”.^81,82^ Naloxone’s effectiveness in reverting synthetic opioid overdoses in the real world is limited by the narrow intervention window. In response to this issue, researchers at the University of Washington have developed a wearable system that administers naloxone after detecting respiratory depression,^83^ but it is an invasive system still in development stage.

Despite its limitations, naloxone remains the primary drug for reversing opioid overdose symptoms, prompting ongoing efforts to find more effective treatments.

### 3.3 Alternative Overdose Prevention Strategies

**Figure 7:**
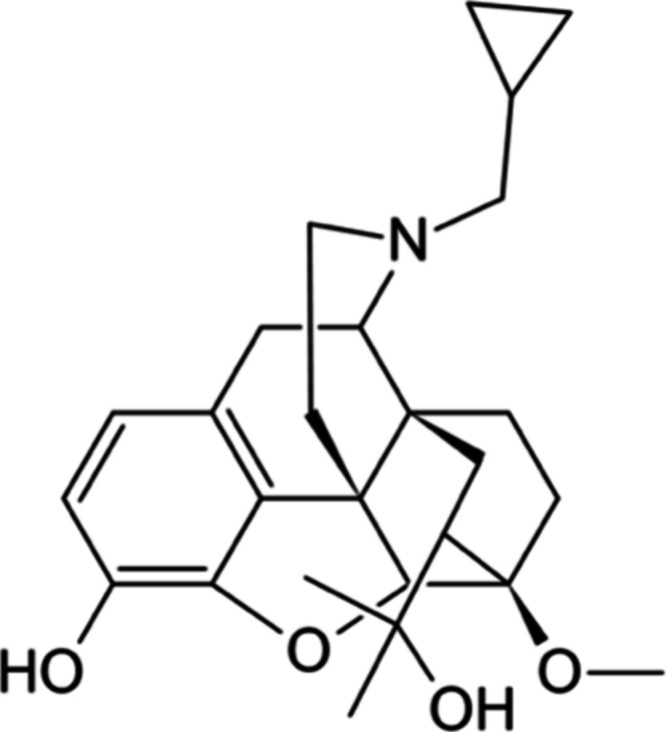
Chemical structure of diprenorphine.

As an alternative to naloxone, other μOR antagonists are available. Naltrexone^84^ and nalmefene^85^ are antagonists characterized by a very similar structure, differing only in position 7: naltrexone features a carbonyl group, while nalmefene contains a methylene group. They share comparable chemical and pharmacodynamic properties. With a significantly longer half-life compared to naloxone (8–10h versus 1–2h), they exhibit enhanced efficacy against long-acting opioids (excluding fentanyl) and demonstrate low bioavailability via intranasal administration. Therefore, they are not considered viable options for addressing fentanyl and derivative overdoses; instead, they are primarily utilized, for instance, in alcohol withdrawal. The exploration of partial agonists, such as buprenorphine^75^ and diprenorphine (Figure 7),^86^ opioid compounds classified under the thebaine derivatives, has also been undertaken.

Diprenorphine, a mild nonselective partial agonist, has demonstrated promising outcomes. Studies have revealed that when naloxone is administered, higher doses are necessary to revert the respiratory depression caused by overdose compared to the one caused by morphine. Interestingly, the same quantity of diprenorphine has been observed to restore normal breathing equally in cases of fentanyl and morphine overdose. The pharmacodynamic rationale behind this phenomenon remains unidentified. It is hypothesized that the increased effectiveness of diprenorphine may arise from its higher lipophilicity in contrast to naloxone, or from a distinct binding pocket orientation within the μ-opioid receptor.

In addition to molecules acting on the opioid receptor, various classes of drugs that stimulate respiratory activity, such as ampakines (AMPA agonists), NMDA antagonists, serotonin or dopamine receptor agonists, phosphodiesterase inhibitors, potassium channel blockers affecting oxygen-sensitive cells of carotid chemoreceptors, and other classes, have been proposed and tested in animal models.^87^ While the results appear promising, preliminary data require more substantial statistical evidence before widespread implementation.

### 3.4 Medications Utilized for Deterrence and Managing Withdrawal Symptoms

To address chronic opioid addiction, which may develop even with the use of synthetic opioids like fentanyl, three pharmacological approaches are employed: substitution medications, nonsubstitution medications, and symptomatic medications. Substitution medications involve opioids with reduced side effects, such as methadone or buprenorphine, administered at decreasing dosages over time.^88^ Nonsubstitution medications encompass substances that generally alleviate symptoms of opioid withdrawal syndrome. Clonidine, traditionally an antihypertensive and α2-adrenergic receptor agonist, has been off-label approved for OUD for years.^89^ Its effectiveness is related to the inhibition of central adrenergic transmission, mitigating the “stress” state induced by opioid withdrawal. Symptomatic medications target individual withdrawal symptoms; for instance, anxiolytics for anxiety, antiemetic or antinausea drugs for nausea, or antidiarrheal medications for diarrhea resulting from substance discontinuation.

The strategies outlined for addressing overdose and addiction, as previously mentioned, are insufficient in combating the current opioid epidemic. Urgent innovative solutions are required to address this crisis effectively, focusing on prevention and treatment.

## 4 New Strategies: Biological Preparations

The concept of developing vaccine solutions against substances of abuse was initially suggested in the 1970s.^90,91^ Initially, the primary goal was not oriented toward therapeutic intervention but rather focused on engineering and developing antibodies for the forensic detection of common illicit substances. More specifically, researchers in the early 1970s dedicated their efforts to the formulation of haptens (small molecules able to elicit immune response) that imitated the structure of molecules like morphine, methamphetamine, and nicotine to provoke an immune response when linked to specific carriers. It was not until after 2000 that the therapeutic potential of this innovation was envisioned, leading to the design of monoclonal antibodies or vaccines aimed at intercepting drugs, impeding their access to the central nervous system, and thereby preventing their recreational and harmful effects.^91^ Initially, interest was directed toward nonopioid substances due to the availability of naloxone as an antidote. However, the inadequacies of naloxone in combating synthetic opioids have recently spurred interest in biological interventions to address the opioid crisis. Various research endeavors in the pharmaceutical, pharmacological, and immunological domains have outlined potential monoclonal antibody treatments (murine, chimeric, and human) and vaccines (utilizing different platforms). The primary pharmacodynamic requirement for these antibodies, whether administered directly or induced *in vivo* through the use of vaccines, is to bind fentanyl with a higher affinity than that of the fentanyl-μOR complex (Kμ = 1.2 nM), thereby sequestering the fentanyl molecules in the bloodstream to impede their passage through the BBB and prevent their interaction with the μOR.

Another crucial pharmacodynamic aspect is the so-called fentanyl pan-specificity: the antibodies should also bind fentanyl derivatives to broaden their effectiveness, particularly since illicitly manufactured fentanyl often includes derivatives like carfentanil to enhance potency inexpensively. However, these antibodies should not bind to other opioids to allow for continued withdrawal therapy for individuals already undergoing addiction treatment.

Besides such prerequisites, these antibodies should be present in high quantities for an extended period without eliciting adverse immune reactions. This approach contrasts with conventional pharmaceutical research, where small molecules are designed to target known macromolecular targets. In this case, the small molecule itself is the target, and macromolecular structures are tailored to interact stably with the small molecule’s structure.

In this context, it must be bared in mind that passive immunity is obtained when the subject is administered antibodies to a disease, while the active one is generally conferred through an immune response generated by the immune system.^92^

Since 2020, numerous studies have explored the development and evaluation of antibodies and vaccines against fentanyl: in the following sections the most significant examples reported in the literature will be analyzed and categorized based on antibody origin, with a focus on pharmacodynamic considerations.

### 4.1 Interaction between Fentanyl and Chimeric Monoclonal Antibodies: An Example

In 2021, Ban et al. reported the development and analysis of chimeric monoclonal antibodies produced by immunizing mice with a vaccine previously created by the same team of researchers.^93^ The vaccine, described in 2020,^94^ comprised a fentanyl hapten with a molecular structure closely resembling that of the original molecule. The only distinction was an amide group located in the *para* position of the benzene ring of the phenylethyl linked to the piperidine, connected to the aromatic ring on the nitrogen side with the carbonyl substituted by a two-carbon alkyl chain ending in a thiol group essential for binding the hapten to immunogenic complexes. These complexes, in this instance, were linked to an inactivated tetanus toxin, capable of triggering an immune response. A similar strategy was pursued, some years before, by the research group of Bremer and colleagues. In this study, a vaccine eliciting high levels of antibodies with cross-reactivity for fentanyl analogues, as demonstrate in mice.^95^

Concerning the hapten, its structural similarity with fentanyl is pivotal for fentanyl pan-specificity, a feature that expands the vaccine’s range of effectiveness to all related molecules while preventing activity toward those used in tapering off.

The immunogenic element (inactivated tetanus toxin, TT) was then conjugated to the para-aminofentanyl hapten using an SM(PEG)2 linker. The efficacy of this vaccine in mice was demonstrated, and the vaccine itself (properly enriched with the necessary components to induce immunological responses) appeared promising. Ban et al. subsequently isolated B lymphocytes reactive to fentanyl from the spleens of immunized mice and fused them with myeloma cells to create murine hybridomas; RNA from these hybridomas was isolated, sequenced, and integrated into linear human vectors containing genes that encode the constant regions of human IgG1. By introducing these vectors into *Escherichia coli* or Chinese hamster ovary cells (CHO-S cells), the desired chimeric antibodies, featuring human constant regions and murine variable segments, were produced.

Then, two antibodies named P1B6H7 and P1C3H9 were selected for an analysis based on their high affinity for fentanyl and fentalogs. *In vitro*, both P1B6H7 and P1C3H9 demonstrated nanomolar affinity for fentanyl, with K_D_ values of 1.28 ± 0.12 nM and 0.15 ± 0.03 nM respectively. The EC_50_ concentrations for these antibodies, representing the dose at which 50% of fentanyl is bound, were determined to be 1.96 nM for P1B6H7 and 1.91 nM for P1C3H9. Regarding fentanyl derivatives such as acrylfentanyl, cyclopropylfentanyl, and furanylfentanyl, P1B6H7 exhibited affinities of 1.21 nM, 7.71 nM, and 2.36 nM, while P1C3H9 showed affinities of 13.89 nM, 2.11 nM, and 8.87 nM, respectively. Notably, a lower concentration indicates a higher potency of the antibody in sequestering the molecule. It is worth mentioning that the antibodies did not exhibit any activity against buprenorphine, methadone, naloxone, or naltrexone during the examination.

*In silico* molecular docking analysis conducted using Chemical Computing Group MOE software unveiled distinct binding modes of fentanyl by the two antibodies. The final model energy was calculated to be −3,546.84 kJ/mol for P1B6H7 and −3,816.92 kJ/mol for P1C3H9 (for fully optimized energy structures), with a root-mean-square deviation (RMSD) of 0.27 Å between the fully optimized structure and the initial model. In P1B6H7, the orthosteric binding site forms a deep pocket, orienting fentanyl with its phenylethyl end facing away from the solvent. Conversely, in P1C3H9, the binding site resembles more of a “groove” on the surface than a pocket yet exhibits significant complementarity. The structural alignment of the variable regions of the two antibodies reveals that the two macromolecules do not align perfectly. Variances are observed in both the heavy chains (with a 42% homology) and the light chains (52%). Notably, differences are most prominent in the CDRs (complementarity-determining regions). For instance, LCDR1 is 5 residues longer in P1C3H9 compared to P1B6H7. All CDR loops contribute to the orthosteric pocket of the P1B6H7 antibody, while only five out of six CDR loops are engaged in forming the P1C3H9 binding site, with LCDR2 lacking direct contact with the ligand. The molecular structures of the fentanyl–antibody binding site exhibit remarkable similarity, with an RMSD of 0.631 Å.

In the instance of P1B6H7, an aspartic acid residue of the heavy chain (Asp101H; the H denotes heavy chain) interacts through the carboxyl group with the carbon at position 1 of the piperidine ring of fentanyl using a hydrogen donor, creating a hydrogen−π bond. The amide nitrogen of fentanyl interacts with the π cloud of the aromatic ring of the tyrosine of the light chain Tyr91L (L = light chain) in a cation−π interaction; the electron cloud of the aromatic ring of the tyrosine is also influenced by the hydrogen atoms of the carbons at positions 2 and 4 of the piperidine ring of fentanyl.

The affinity with P1C3H9 is higher: in this case, the amide nitrogen of fentanyl engages with the electron cloud of the aromatic ring of a tyrosine (Tyr102H) through a cation−π interaction. Furthermore, the hydrogen atom of carbon 4 of the piperidine of fentanyl interacts with the same aromatic ring via a nonpolar hydrogen−π interaction. Tyr102H also interacts with the piperidine nitrogen and with aniline. Additionally, the π cloud of the aromatic phenylethyl ring of fentanyl interacts with the hydroxyl group of tyrosine 102H through a nonpolar π–hydrogen interaction. The higher preference of P1C3H9 for fentanyl stems from the interactions facilitated by the two aromatic rings, which lack in the case of P1B6H7. The overall structure of fentanyl appears more condensed and less elongated. Both antibodies engage in cation−π interactions with the amide nitrogen of fentanyl through tyrosine. While P1B6H7 features a deeper binding pocket, P1C3H9 exhibits stronger interactions. Consequently, fentanyl shows a slightly higher affinity for P1C3H9; nonetheless, P1B6H7 also demonstrates a favorable affinity, with a K_D_ in the nanomolar range. The robust mAb–fentanyl affinity correlates with effective outcomes observed in murine models: mice treated with these mAbs displayed altered fentanyl distribution, resulting in diminished analgesic effects. Notably, only 1/3 of the initial 0.1 mg/kg dose reached the CNS, with the majority being sequestered by the mAbs in the bloodstream. Further clinical trials are imperative to validate both efficacy and safety profiles.

### 4.2 Interaction between Fentanyl and Human Monoclonal Antibodies: An Example

Human or humanized antibodies are more widely accepted by human organism than chimeric antibodies.

An example of engineered human monoclonal antibodies targeting fentanyl and its analogues was recently reported.^96^ The approach involved immunizing rats with a specific immunoconjugate, isolating antigen-specific B lymphocytes, cloning them in cell lines capable of producing the desired antibodies, and ultimately obtaining the intended monoclonal antibodies. A unique immunoconjugate, utilizing a hapten derived from carfentanil instead of fentanyl, was employed as a vaccine along with a suitable adjuvant. The vaccine was administered to rodents through a series of spaced injections. To identify B lymphocytes capable of generating antibodies against fentanyl and its analogues, probes containing both fentanyl and carfentanil were utilized. Following the selection of B lymphocytes and cloning in human mAb expression vectors, HEK-293 cells were infected with the genetic material to produce the antibody pool. Among the obtained antibodies, the analysis revealed that the most promising candidate was an antibody named P1A4, exhibiting exceptional association rate constant (*k*_a_) and dissociation rate constant (*k*_d_) values, along with a high affinity for fentanyl and carfentanil. P1A4 was then further enhanced by modifying three amino acid residues to improve its properties, resulting in a mAb named C10–S66K. The binding characteristics of fentanyl to C10–S66K show affinities typically surpassing those to the μ-opioid receptor and similar to the mAbs examined in the previous section. The elevated *k*_a_ value demonstrates the rapid formation of the antibody-ligand complex, while the low *k*_d_ and K_D_ values underscore the challenge in separating the two components, indicating a strong affinity.

**Figure 8:**
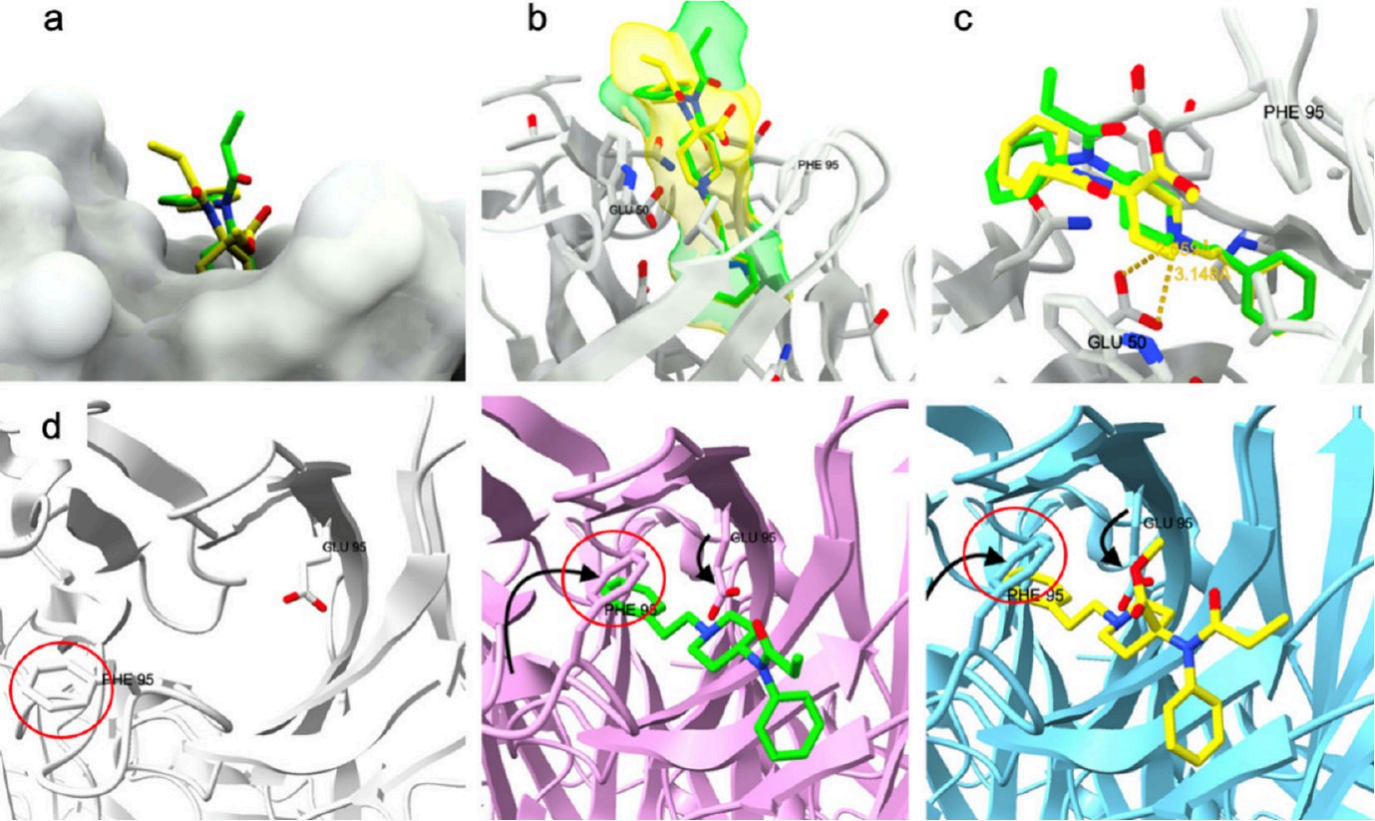
a: Side view of the binding pocket of fentanyl–C10–S66K (green; PDB ID: 8TFQ) and carfentanil–C10–S66K (yellow; PDB ID: 8TFP). b: Different orientation of the amide carbonyl. c: Salt bridge between the piperidine nitrogen Glu50H. d: Comparison of the binding pocket between C10–S66K without ligand (left; PDB ID: 8TFR) and with ligand (with fentanyl, in the center, in green and C10–S66K in purple, and with carfentanil, on the right, in yellow with C10–S66K in light blue), with the movement of the residues Phe95L and Glu95H highlighted.

In further examination of the pharmacodynamic aspects, the binding orientations of fentanyl (PDB ID: 8TFQ) and carfentanil (PDB ID: 8TFP) at the orthosteric binding site of C10–S66K were assessed through X-ray crystallography (resolved at 1.80 Å for fentanyl–mAb binding, 1.78 Å for carfentanil–mAb binding, and 2.96 Å for ligand-free C10–S66K; PDB ID for the apo structure: 8TFR). It was revealed that C10–S66K predominantly interacts with fentanyl and carfentanil via the phenylethyl group and the piperidine ring present in both compounds. These components are deeply situated within the binding cavity and form the essence of the interaction. The binding site, akin to a “lock,” is characterized by narrow boundaries delineated by the aromatic side chains of Phe95L and Trp34H. The interaction is primarily driven by van der Waals interactions, and a salt bridge is established between the carboxyl group of a glutamic acid residue on the heavy chain (Glu50H) and the piperidine nitrogen of both substances. The negatively charged nature of the binding site enhances favorable electrostatic interactions with fentanyl (pKa = 8.12) and carfentanil (pKa = 8.05), both of which exhibit protonated nitrogens at physiological temperatures. The conformations of the complementarity determining regions (CDRs) in the fentanyl–C10–S66K and carfentanil–C10–S66K complexes are nearly identical: C10–S66K employs the same amino acid residues to bind both fentanyl and carfentanil. The main difference in the positioning of the two molecules lies in the spatial arrangement of the amide group (Figure 8, panels a, b and c): in carfentanil, the amide carbonyl assumes a slightly different orientation, interacting with Tyr52H. This variance is attributed to the steric hindrance caused by the methoxycarbonyl group attached to the carbon at position 4 of the piperidine, which displaces the amide carbonyl to a more distant location without introducing any additional interactions with the mAb binding site.

A comparison of the structures of C10–S66K without ligand (Figure 8, panel d) and with fentanyl and carfentanil bound reveals conformational differences in two residues: Glu95H and Phe95L. The side chain of Glu95H shifts slightly inward to accommodate the phenylethyl group of fentanyl and carfentanil, while the drug pocket appears larger in the unliganded structure due to a different conformation of Phe95L. In the drug-binding structures, the side chain of Phe95L rotates to engage the piperidine ring of fentanyl and carfentanil. This analysis suggests that Phe95L is flexible, indicating that the pocket adopts a more open conformation without a ligand and closes upon ligand binding. As demonstrated, C10–S66K primarily interacts with the phenylethyl and piperidine core of fentanyl and carfentanil, enabling the antibody to bind to various fentanyl analogs with a similar structure. Molecular docking simulations have revealed the binding orientations of certain fentanyl derivatives. These simulations show a consistent alignment of molecules, with the phenylethyl group positioned deep within the binding pocket, and minor conformational differences in functional groups extending from the amide nitrogen. The flexibility of the C10–S66K pocket allows for the accommodation of different moieties, such as the ethylcyclopentane of sufentanil and the phenylethyl methyl group of α-methylfentanyl. Conversely, additional functional groups extending from the piperidine ring of alfentanil and remifentanil, directed toward the pocket wall opposite to the methoxycarbonyl group of carfentanil, create unfavorable steric hindrance. These distinctive features of alfentanil and remifentanil, along with variations in the phenylethyl ring, may contribute to their lower binding affinity. While some derivatives have functional groups extending from the amide nitrogen that could potentially clash with the CDRH2 domain in the C10–S66K binding site, their observed affinities suggest that CDRH2 can adapt to different conformations to accommodate these derivatives effectively. In vivo testing was not conducted on the complete antibody but on the more tolerated single-chain variable fragment (scFv) derivative, which was enhanced by fusing it with an albumin binding domain (ABD) to prolong its plasma half-life. The study focused on evaluating its effectiveness in reversing carfentanil overdose in mice. The findings demonstrated superior efficacy compared to naloxone, particularly in maintaining a consistent restoration of respiratory function over an extended period, and, unlike naloxone, which can lead to a respiratory distress shortly after its peak of efficacy, repeated doses were not needed.

### 4.3 Interactions between Fentanyl and Vaccine-Induced Antibodies: Two Examples

In 2023, two models of binding pose analysis of fentanyl complexed with antibodies resulting directly from the administration of an immunoconjugated vaccine were reported: Rodarte et al. scrutinized the fentanyl–antibody interactions in 2023, examining a vaccine containing keyhole limpet hemocyanin KLH, an immunogenic protein;^97^ in the same year, Triller et al. introduced a highly innovative approach, utilizing a trypanosome as an immunogenic agent to produce antibodies.^81^

Rodarte et al. employed a vaccine developed by the same team of researchers, comprising a fentanyl hapten with modifications including a glutaric acid monoamide replacing phenylethylbenzene, connected to the ethyl group via the amide nitrogen. Additionally, the second carboxylic group was substituted with a linker of four glycines, all conjugated to hemocyanin, the immunogenic element.^98^ The effectiveness of this immunoconjugate, denoted as F-sKLH, was demonstrated *in vivo*.

**Figure 9:**
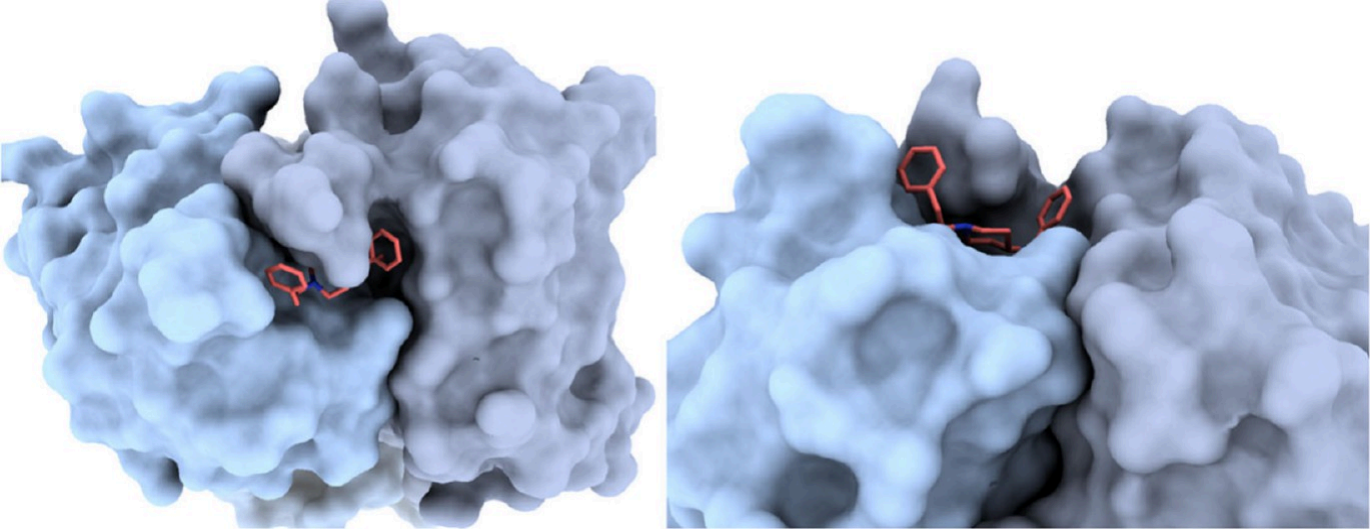
Top (left) and cross-sectional (right) views of the binding pose of fentanyl (red) in HY6-F9.

**Figure 10:**
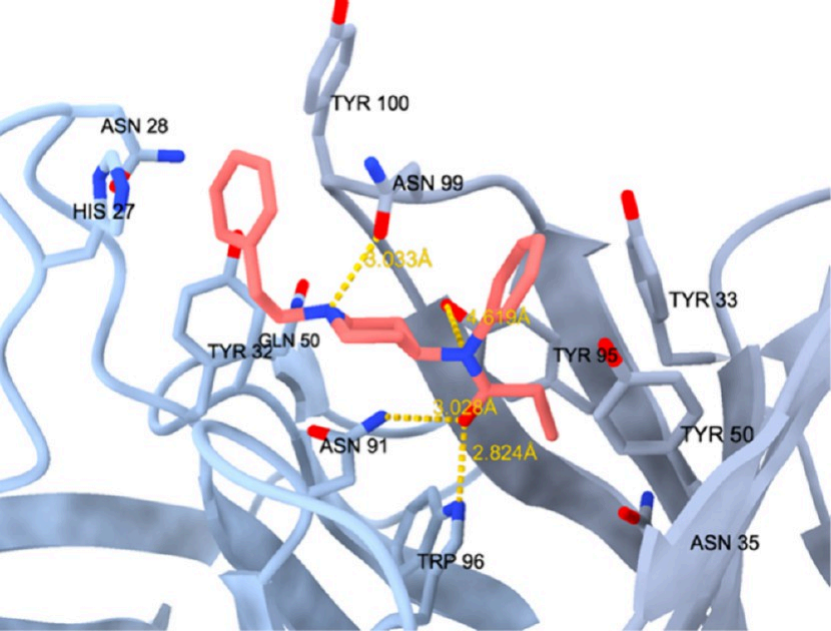
Fentanyl-HY6-F9 bond.

Mice immunized with the vaccine yielded the HY6-F9 antibody, which was then isolated. The fentanyl–HY6-F9 complex was structurally determined at 1.75 Å resolution through X-ray crystallography, marking the first fentanyl–antibody structure to be deposited in the PDB (PDB ID: 7U64). A remarkably high binding affinity (K_D_ = 5.0 ± 1.2 pM) was observed, with a *k*_a_ = (1.4 ± 0.01) × 10^5^ 1/Ms and a *k*_d_ = (6.8 ± 1.6) × 10^–6^ 1/s. Figure 9 illustrates the top and section views of the binding pocket, and Figure 10 depicts the interaction pattern.

The deep binding pocket accommodates approximately 85% of the molecular structure of fentanyl, with the amide group side serving as the entry point. The phenylethyl component remains more exposed on the surface. CDRH3 envelops the central fentanyl region from above, facilitated by a network of hydrogen bonds among CDRL1, CDRL2, and CRDH3 residues. A critical interaction occurs with Asn99H, forming a salt bridge with the protonated tertiary amine of the piperidine ring. The fentanyl amide ketone exhibits dual behavior, potentially binding to Asn91L or Trp96L via hydrogen bonds. This interaction induces a partial positive charge on the adjacent carbon, enabling a π-cationic interaction with Tyr95H’s aromatic ring. The *N*-phenyl and propionyl groups fit into a small hydrophobic pocket, with the propionyl group penetrating deeper. An intriguing π-π interaction may form between the phenethyl ring of fentanyl and His27L.

Additionally, as anticipated, Triller et al. have developed a unique vaccine that utilizes a distinctive property of *Trypanosoma brucei*. Instead of a traditional immunoconjugate, the vaccine leverages the dense and repetitive pattern of glycoproteins, known as variant surface glycoproteins (VSG), displayed on the surface of the *Trypanosome*. These VSGs, found in approximately 10 million identical copies on a single trypanosome, induce the host to mount a precise and enduring immune response. Trypanosomes possess the ability to switch from one type of VSG to another to evade the host’s immune defenses. By engineering the VSGs with fentanyl haptens and eliminating the ability to switch VSGs, the vaccine induces the production of specific and long-lasting antibodies against fentanyl.

Conjugation of the fentanyl hapten with VSGs involved the insertion of sequences sensitive to sortases, enzymes with transpeptidase activity, into the haptens themselves. The VSGs had been previously modified to be responsive to this process, known as sortagging. The fentanyl hapten was synthesized following the pathway that commenced with *N*-phenyl-N-[1-(2-aminoethyl)piperidin-4-yl]propanamide, differing from fentanyl by having an amino group in place of the phenyl connected to the piperidine nitrogen via the ethyl group. Through a reaction with the monomethyl ester of glutaryl chloride in the presence of pyridine, the mentioned amine was transformed into an amide. The methyl ester was converted from O-Me to O–Li by reacting with LiOH. Subsequently, solid-phase peptide synthesis (SPPS) facilitated the conjugation of a peptide segment to the fentanyl structure on the ester side. Depending on the amino acids introduced in the reaction, two derivatives were produced: Fen-G4 (comprising four glycines, solely utilized for pharmacodynamic analysis of antibody binding) and the “Fen-sort” (linked to an amino acid chain GGGSLPSTGG), which is the fentanyl hapten responsive to sortase activity (the sequence LPSTGG is recognized by sortases). It is worth noting that Fen-sort lacks the benzene ring present in the fentanyl structure, replaced by an ethyl group. Specifically, Fen-sort is employed in the proposed vaccine: the sortase cleaves the bond between threonine and the two glycines, facilitating the attachment of the peptide to the trypanosomal VSGs previously engineered to accommodate the specific peptide structure, carrying the fentanyl moiety.

Upon obtaining inactivated trypanosomes coated with fentanyl haptens, the vaccine was administered to murine animal models, initially followed by a booster dose after a period of time. Although the initial antibody production was lower compared to other immunoconjugates, a substantial and sustained increase in antibody production was observed postbooster, lasting for at least 4 months. This extended efficacy indicated the presence of memory cells, affirming the enduring effectiveness of the trypanosome-based vaccine formulation. The protective efficacy of the fentanyl-induced antibodies was subsequently demonstrated through the hot plate test and Straub’s tail test. Administering 100 μg/kg of fentanyl, a pharmacologically active dose, did not exhibit the typical analgesic effects in both tests. To validate the efficacy of the antibody in sequestering fentanyl at the serum level, fentanyl concentrations in the central nervous system and plasma of vaccinated mice and control subjects were measured. Vaccinated mice showed fentanyl levels below 5 ng/mL in the CNS and between 80 and 280 ng/mL in the plasma, while control animals exhibited 10–20 ng/mL in the CNS and less than 5 ng/mL in the plasma. Subsequent analysis involved selecting B lymphocytes capable of producing fentanyl-specific antibodies, cloning them, and expressing the BCR as recombinant mAbs. The affinity properties of the obtained mAbs were further examined using isothermal titration calorimetry (ITC), revealing fentanyl-antibody affinity in the nanomolar range (0.7–1 nM) with exothermic binding reactions. Despite a decrease in entropy, the system’s free energy was reduced due to a decline in enthalpy. Pharmacological assessments were conducted to validate the efficacy of the generated antibodies. Following the selection of FenAb136, the antibody with the lowest fentanyl affinity among the purified antibodies, pharmacological tests were performed. Administration of FenAb136 into animal subjects, followed by fentanyl administration, confirmed the presence of complexed fentanyl in the plasma through liquid chromatography–mass spectrometry (LC-MS), demonstrating dose-dependent protection from fentanyl. The potential use of these mAbs, particularly FenAb136, as a postoverdose treatment was explored. Mice were injected with a lethal dose of fentanyl, followed by intravenous administration of FenAb136 and naloxone. The monitoring the activity of the animals revealed a comparable efficacy with respect to naloxone, with FenAb136 exhibiting a faster reversal of respiratory depression than the above-mentioned drug, showcasing its rapid action postadministration.

**Figure 11:**
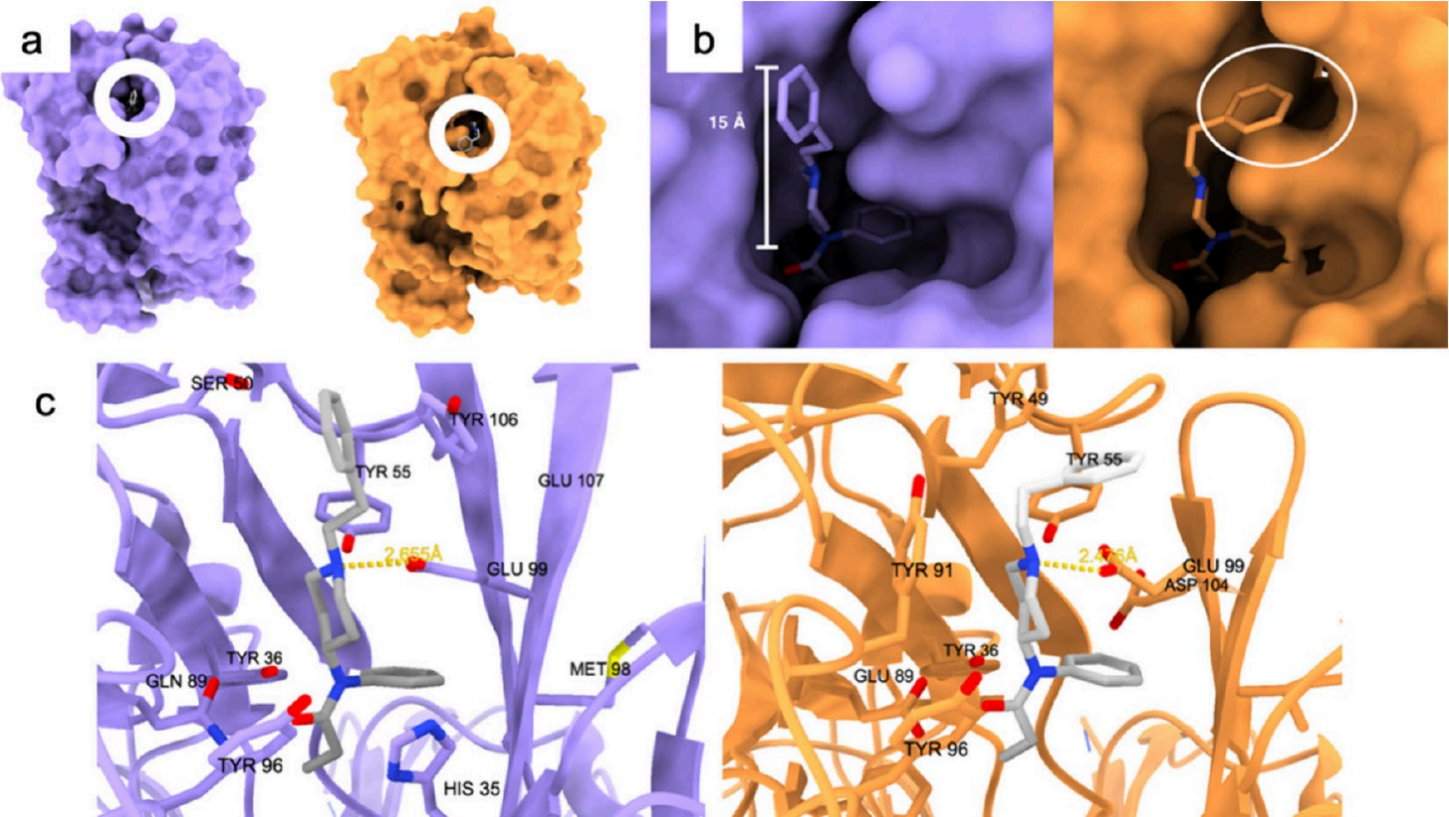
a: lateral perspectives of fentanyl bound by FenAb609 (purple, left) and FenAb208 (orange, right). b: fentanyl binding site for FenAb609 and FenAb208; the hydrophobic pocket encompassing the phenylethyl in FenAb208 was highlighted. c: close-up of fentanyl binding to FenAb609 and FenAb208.

The antibodies FenAb609 and FenAb208 were selected for pharmacodynamic analysis of fentanyl binding. X-ray crystallography solved the structures of the fentanyl–FenAb609 and fentanyl–FenAb208 complexes (Figure 11; PDB ID: 7QT3, 7QT2). These mAbs share a common binding mechanism with a deep orthosteric pocket formed by residues from both heavy and light immunoglobulin chains. For instance, the binding of fentanyl to FenAb609 is a clear example of this mechanism, which is also applicable to FenAb208. The pocket, approximately 15 Å deep, is formed by CDR regions at the top and β-sheet residues at the bottom. Fentanyl adopts an elongated conformation within the cavity, with only the phenylethyl group slightly protruding. In FenAb208, the shortened structure of the CDR3 domain creates a hydrophobic recess for the phenylethyl group. Despite the absence of this group in the hapten, effective complexation by the antibodies is demonstrated. The molecule is securely held in the pocket by hydrophobic and van der Waals interactions, as well as a salt bridge, anchoring the piperidine ring. The *N*-phenyl-propanamide group engages in various interactions, with the *N*-phenyl ring fitting into a specific cavity and the propionyl group forming hydrogen bonds and hydrophobic interactions.

The three-dimensional conformation of these mAbs likely undergoes significant changes in the presence of the ligand, such as fentanyl, resulting in a reduction of the degrees of freedom and entropy within the structure due to a higher order. Despite this, it has been theorized that the ligand-antibody bond may lead to an entropy increase due to the desolvation of amino acid residues involved in the interaction.^99^ The high affinity observed, as evidenced by crystallography, is attributed to multiple interactions between fentanyl and the mAb, including two ionic bonds, and a well-suited binding pocket for fentanyl. However, these interactions are highly specific for the fentanyl structure. Research indicates that antibodies generated from immunization with this vaccine may not be as effective against fentanyl derivatives, potentially limiting their field of application and eventually pushing illicit markets toward the trading of derivatives like carfentanil.^100^ Nevertheless, the innovative approach taken in formulating this preparation and the promising results achieved position it among the most intriguing solutions proposed thus far.

### 4.4 Comparison between Fentanyl–Antibody Binding and Fentanyl−μOR Interaction

The understanding of fentanyl-μ-opioid receptor interactions from a structural point of view were of guiding relevance in the design of haptens and in understanding the binding of this compound and its analogues to antibodies. In this Perspective, the binding of fentanyl to μ-opioid receptor was discussed in depth in section 2.4, while the binding motif with antibodies and vaccine-induced antibodies was described in sections 4.2 and 4.3, respectively. In this section, a comparison between fentanyl–antibody binding and fentanyl−μOR interaction will be presented.

The interactions between fentanyl and antibodies discussed thus far exhibit certain similarities and a shared binding pattern can be observed. As previously mentioned, the presence or absence of phenylethylbenzene in the hapten utilized determines the orientation of the fentanyl molecule within the orthosteric pocket of the antibody. When the aromatic ring is present, such as in mAbs P1B6H7 and P1C3H9, or C10–S66K, the fentanyl molecule adopts the FPA conformation. This conformation can be considered as opposite to the above-mentioned APF, in which the amide group points toward the inner part of the pocket. In the FPA pose, it is the phenyl ring that is buried within the binding site. Conversely, antibodies like HY6-F9, FenAb609, and FenAb208, generated from vaccines without benzene in the hapten, accommodate the amide portion of fentanyl deeply in the orthosteric pocket, leading to the APF conformation. Antibodies binding fentanyl in the FPA pose exhibit lower affinity but broader activity toward derivatives. In contrast, those binding in the APF pose have higher affinities but may be less effective against derivatives with modifications, particularly in the amide portion. Structural similarities exist among all antibodies, with varying heavy and light chain similarities. The binding of fentanyl to antibodies typically involves a salt bridge interaction with the tertiary amine of the piperidine ring. Different residues, such as glutamate, asparagine, or glutamine, drive this interaction in various antibodies. The bond with the piperidine nitrogen, found in the fentanyl-μ-opioid receptor complex, is also crucial, with the tertiary amine interaction being fundamental. In the μOR, the interaction involves an aspartic acid residue, belonging to the polar DQY motif, leading to delocalization and weaker intensity compared to antibodies. Antibodies exhibit discrete,^100^ separated bonds, making them more resistant to dissociation. Weak interactions in the μOR contrast with the intense π-polar or π-π interactions in antibodies, showcasing significant electrostatic complementarity and hydrophobic region accommodation. Disparities in amino acid composition between antibodies and the μOR derive from their distinct biological origins, as antibodies derive from B cell receptors and the μOR from a GPCR. Despite these differences, both macromolecules interact with fentanyl and its derivatives through the tertiary amine. The absence of a delocalization network in the bond between the amine and antibody residues, along with more intense hydrophobic interactions, elucidates why fentanyl exhibits greater affinity and lower dissociation rates for antibodies compared to the μOR. It is crucial to emphasize that the antibodies should bind not only fentanyl but also its derivatives, while maintaining selectivity for this chemical class of compounds. Among the tested candidates, P1B6H7, P1C3H9, and C10–S66K were examined for their ability to bind fentalogs. The results indeed confirmed that these antibodies can bind not only fentanyl but also a variety of phenylpiperidines. The structural variances in the haptens used to produce the antibodies (such as modifications to the phenylethyl and piperidine portions of the fentanyl molecule) influence the binding mode. More specifically, chirality does not seem to significantly impact binding, as even though some fentanyl derivatives may exhibit chirality at carbon 4 of the piperidine ring, this does not appear to affect the interaction with the antibody target.

### 4.5 Perspectives and Challenges for Monoclonal Antibodies and Vaccines Against Fentanyl and Derivatives: Considerations for the Future

The development of antibodies and vaccines against fentanyl unavoidably carries chances and challenges. The translation of preclinical results into real world applications must take into account pharmacodynamic and pharmacokinetic aspects, ranging from efficacy to selectivity and considering formulation stability.^101,102^

In the past years, several clinical trials have been conducted on therapeutic biological macromolecules as remedies against small molecules. Most of these trials focused on vaccines against nicotine, such as NicVax,^103^ NIC-002,^104^ and TA-NIC.^105^ Unfortunately, none of these vaccines progressed beyond phase III of clinical development due to unsatisfactory results. Despite showing promising data *in vitro* and in preclinical tests, human trials did not meet expectations. It must be anyway pointed out that nicotine differs pharmacodynamically and pharmacokinetically from other drugs, primarily due to its lower potency. While nicotine vaccines could produce antibodies that bind to the substance, they were not in a sufficient quantity to be effective. On average, these antibodies could only bind the equivalent amount of nicotine found in a single cigarette, rendering them ineffective in combating addiction.^97^ However, these vaccines had minimal side effects. Despite the setbacks with nicotine vaccines, research in this area persists. An anticocaine monoclonal antibody (mAb) called h2E2 is in the final stages of preclinical development.^106^ Additionally, the anticocaine vaccine dAd5GNE is in phase I of clinical development.^107^ An antimethamphetamine mAb known as IXT-m200 is nearing completion of phase II clinical trials.^108^ Furthermore, the oxycodone vaccine OXY-KLH, designed to combat heroin and morphine as well, is also in phase II trials.^109^ This vaccine was the first developed against opioids in 2014, targeting oxycodone, the primary opioid of abuse at that time.^110^

The inefficacy of nicotine vaccines should not obstacle the development of such tools for potent opioids like fentanyl and its derivatives, which are well-suited for binding by monoclonal antibodies or vaccine-induced antibodies due to the minimal amount required for an effective dose, as testified by recent reports.^111,112^ Indeed, *in vitro* studies have shown the effectiveness of these approaches, with promising candidates being the mAb C10–S66K and the trypanosome-based vaccine. Another notable vaccine developed by the University of Houston, though lacking pharmacodynamic studies, shows promise for clinical trials in the near future.^113^ The researchers that led this study are confident to begin clinical trials soon and to bring this vaccine on the market within the next five years.^114^ The antibody CSX-1004, after showing promising results in mouse and monkey models,^111^ approached clinical trials with a “randomized, double-blind, single ascending dose study, designed to assess the safety, tolerability, and PK of a single CSX-1004 injection” (ClinicalTrials.gov ID NCT06005402). While the lack of homogeneous data impairs the full and direct comparison of the results available for different studies, as several researches are still ongoing, an overview of the main preparations currently under investigation is reported in Table 1.

**Table 1:**
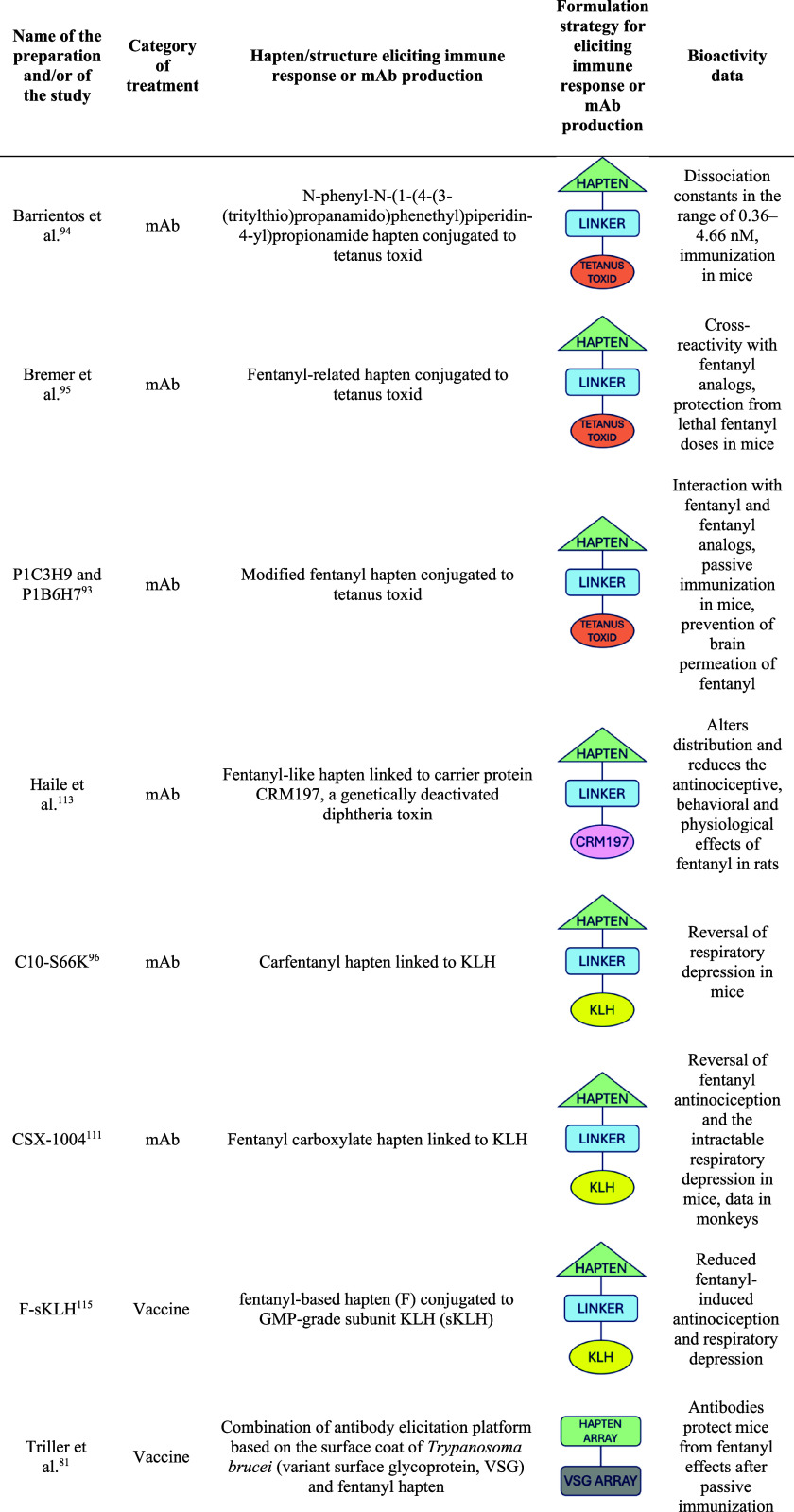
Overview of the Preparations Described in the Perspective and Currently under Investigation

One of the crucial aspects that must be considered in the development of monoclonal antibodies and vaccines is their pharmacokinetic profile. This aspect is currently being studied from the preclinical and clinical point of view. Some data are available concerning the properties of hapten bioconjugates. More specifically, the fentanyl-CRM preparation was administered in 3 doses (1 “vaccine” and 2 “boosters”) separated by 3 weeks in mice.^113^ On the other hand, as anticipated above, the pharmacokinetic properties of the antibody CSX-1004 after single injection are currently being investigated in clinical trials.

Concerning indications, while monoclonal antibodies are envisioned for regular treatment in high-risk individuals, questions persist regarding the optimal vaccination strategy, including target patient populations and potential expansion beyond addiction history to encompass genetic or environmental risk factors. The prospect of broadening vaccine administration to a wider population hinges on further advancements. An approach could be represented by multivalent formulations for various drug vaccines.^116^ While vaccines offer prophylactic benefits, they may not be suitable for immunocompromised patients: therefore, a dual focus on both vaccines and monoclonal antibodies is crucial. Concerns about individuals switching to other substances highlight the need for versatile remedies capable of addressing diverse addictions. Studies suggest that transitioning from opioid abuse to other substances is less common than anticipated. Furthermore, the risk of escalating opioid doses to bypass antibody effects underscores should not be as frequent, because such practices require high costs. Another concern regards the potential need to administer potent analgesics to vaccinated individuals. This issue can be mitigated by utilizing opioid compounds like buprenorphine, which are unlikely to be targeted by the antibodies produced. Patient adherence poses another significant question; nevertheless, with a well-executed promotional strategy and careful selection of recipients, this should not pose a challenge. Moreover, since the therapy would involve fewer administrations compared to alternative treatments, community skepticism should dissipate in light of an effective remedy with minimal side effects. While uncertainties persist, certain key aspects are consistently observed across various cases. Notably, the high pharmacodynamic specificity toward abused substances while sparing withdrawal medications ensures continuity for the deaddiction treatments, and this should ensure a higher efficacy of this dual strategy, particularly against fentanyl opioids. The efficacy of these antibodies is supported by preclinical trials not only on mice, as already mentioned, but also on other animal models (such as *Macacus rhesus* monkeys),^117^ and results in the elimination of psychotropic and pharmacological effects of fentanyl and analogues. Additionally, adverse reactions seem to be minimal. The optimal formulation of vaccines and mAbs, as well as achieving higher efficiency, remains to be elucidated, possibly through the use of adjuvants like those targeting TLR4 or TLR7/8.^118^ Eventually, the evidence on the possibility of combining the use of monoclonal antibodies against fentanyl and naloxone, testified by the positive results obtained in rat models in terms of faster reversion of respiratory depression, further sustains the potential of this approach.^119^

## 5 Current Treatment of Overdose and Dependence

In conclusion, further advancements are required to validate these solutions for human application. Clinical trials are imperative to confirm the promising outcomes observed in preclinical studies. Understanding the interaction between fentanyl, its derivatives, antibodies produced by vaccines or monoclonal antibodies, and the specific hapten utilized to create these antibodies, along with comparing it to the bond with the μ-opioid receptor, is crucial for translating this knowledge into potent solutions against the opioid crisis.

On the other hand, other aspects must be considered when going through this path. Besides the results of clinical trials and the limitations coming from immune variability, economic feasibility and societal acceptance represent primary challenges for these new therapeutic approaches. In this connection, Weitzman and colleagues very recently examined the potential pitfalls related to the development of such novel tools. A first problem may consist, very simply, in the limited awareness on the topic by the public and biomedical professionals. More precisely, the authors reported the main issues that may affect development and use, which can be clustered into 4 main themes: “(1) population health and healthcare patterns; (2) historical insights and frameworks relevant to optimizing benefits and minimizing harms; (3) vaccine confidence, acceptability, and decision-making; (4) operational issues related to promotion and distribution”.^120^

Thus, the fentanyl-antibody interaction approach represents an innovative, promising and discussed strategy. Undoubtedly, it represents an approach deserving attention, as it potentially paves the way for novel remedies to halt the escalating emergency and address the inadequacy of current antidotes in combating the pressing issues of opioid epidemic.
